# Orphan nuclear receptors-induced ALT-associated PML bodies are targets for ALT inhibition

**DOI:** 10.1093/nar/gkae389

**Published:** 2024-05-16

**Authors:** Venus Marie Gaela, Hsuan-Yu Hsia, Nithila A Joseph, Wan-Yi Tzeng, Pin-Chao Ting, Yi-Ling Shen, Chia-Tsen Tsai, Thomas Boudier, Liuh-Yow Chen

**Affiliations:** Molecular and Cell Biology, Taiwan International Graduate Program, Academia Sinica and Graduate Institute of Life Sciences, National Defense Medical Center, Taipei 11529, Taiwan; Institute of Molecular Biology, Academia Sinica, Taipei 11529, Taiwan; Institute of Molecular Biology, Academia Sinica, Taipei 11529, Taiwan; Institute of Molecular Biology, Academia Sinica, Taipei 11529, Taiwan; Institute of Molecular Biology, Academia Sinica, Taipei 11529, Taiwan; Insitute of Molecular and Cellular Biology, National Taiwan University, Taipei 106319, Taiwan; Institute of Molecular Biology, Academia Sinica, Taipei 11529, Taiwan; Department and Graduate Institute of Microbiology and Immunology, National Defense Medical Center, Taipei 11490, Taiwan; Institute of Molecular Biology, Academia Sinica, Taipei 11529, Taiwan; Molecular and Cell Biology, Taiwan International Graduate Program, Academia Sinica and Graduate Institute of Life Sciences, National Defense Medical Center, Taipei 11529, Taiwan; Institute of Molecular Biology, Academia Sinica, Taipei 11529, Taiwan; CENTURI multi-engineering platform, Aix-Marseille Université, Marseille 13288, France; Molecular and Cell Biology, Taiwan International Graduate Program, Academia Sinica and Graduate Institute of Life Sciences, National Defense Medical Center, Taipei 11529, Taiwan; Institute of Molecular Biology, Academia Sinica, Taipei 11529, Taiwan

## Abstract

Orphan nuclear receptors (NRs), such as COUP-TF1, COUP-TF2, EAR2, TR2 and TR4, are implicated in telomerase-negative cancers that maintain their telomeres through the alternative lengthening of telomeres (ALT) mechanism. However, how telomere association of orphan NRs is involved in ALT activation remains unclear. Here, we demonstrate that telomeric tethering of orphan NRs in human fibroblasts initiates formation of ALT-associated PML bodies (APBs) and features of ALT activity, including ALT telomere DNA synthesis, telomere sister chromatid exchange, and telomeric C-circle generation, suggesting de novo ALT induction. Overexpression of orphan NRs exacerbates ALT phenotypes in ALT cells, while their depletion limits ALT. Orphan NRs initiate ALT via the zinc finger protein 827, suggesting the involvement of chromatin structure alterations for ALT activation. Furthermore, we found that orphan NRs and deficiency of the ALT suppressor ATRX-DAXX complex operate in concert to promote ALT activation. Moreover, PML depletion by gene knockout or arsenic trioxide treatment inhibited ALT induction in fibroblasts and ALT cancer cells, suggesting that APB formation underlies the orphan NR-induced ALT activation. Importantly, arsenic trioxide administration abolished APB formation and features of ALT activity in ALT cancer cell line-derived mouse xenografts, suggesting its potential for further therapeutic development to treat ALT cancers.

## Graphical Abstract

**Graphical Abstract:**
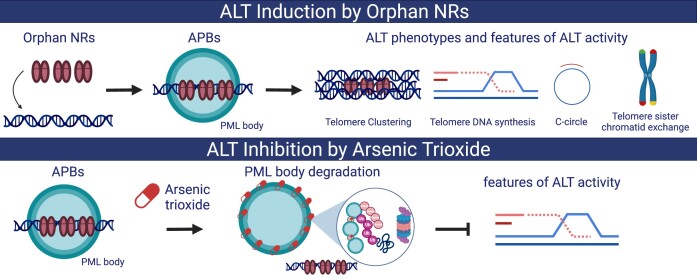

## Introduction

Telomeres are protective nucleoprotein structures at chromosome termini that prevent activation of the DNA damage response and chromosomal fusion. In humans, telomeres consist of 5′ TTAGGG 3′ DNA sequence repeats and telomere-binding proteins of the shelterin complex (1,2). Due to incomplete replication of the lagging DNA strand, telomeres shorten after each round of cell division, termed the ‘end-replication problem’. When telomeres become too short to maintain chromosome integrity and genome stability, cells enter replicative senescence and death (3,4). Cancer cells overcome this problem by activating a telomere maintenance mechanism for their unlimited proliferation. In ∼85% of cancers, telomere length is maintained by reactivating telomerase. However, ∼15% of cancers, particularly in tumors of mesenchymal or neuroepithelial origin, utilize the alternative lengthening of telomeres (ALT) mechanism for telomere maintenance, independent of telomerase. The ALT pathway is a homologous recombination-based telomere maintenance mechanism relying on break-induced replication (BIR)-mediated ALT telomere DNA synthesis (5–7). However, the initiating events that lead to activation of the ALT pathway remain unclear.

ALT cancer cells exhibit associations between the promyelocytic leukemia nuclear bodies (PML-NBs) and telomeres (8), forming the ALT-associated PML-NBs (APBs). PML-NBs are composed of the structural proteins PML and SP100, as well as associated proteins such as DAXX and SUMO. PML-NBs assemble via multivalent SUMO-SUMO-interacting motif (SIM) interactions, which may depend on liquid-liquid phase separation (LLPS) (9,10). APB formation can be promoted by replication stress, with DNA damage agents and loss of several replication stress response proteins increasing APBs in ALT cells (11–14). Formation of APBs is regulated by SUMO modifications on the shelterin components TRF1 and TRF2, with mutations of the sumoylation sites preventing APB formation (15). Aside from their PML-NB components, APBs also contain recombination and repair proteins, supporting that APBs are sites for homologous recombination where a self-perpetuating loop of ALT activity may occur (16–18). Knockdown or knockout of PML in the U2OS ALT cell line reduced recombination-mediated telomere synthesis, suggesting that APBs are critical for ALT activity (7,19). However, APB induction is insufficient to induce ALT telomere DNA synthesis in non-ALT cells (18), indicating that additional factors are important for ALT activation. In addition to APB formation, spontaneous telomere DNA damage response and accumulation of extrachromosomal telomere repeat DNA are ALT characteristics that may arise from and positively regulate ALT activation (11,20–22).

Genetic alterations may underlie ALT development. The most common mutations reported in ALT cancers and cell lines are mutations in genes encoding for α-thalassemia/mental retardation syndrome X-linked (ATRX) and death-domain-associated protein (DAXX) (23–26). Together, ATRX and DAXX form a histone chaperone complex that deposits histone variant H3.3 on telomeres and other heterochromatic regions (27–30). ATRX and DAXX are known to be ALT suppressors, as their re-expression suppresses ALT phenotypes (24,31,32). An *in vitro* cell immortalization study revealed that spontaneous loss of ATRX is an early event during ALT activation (31). ATRX deficiency results in the accumulation of RNA:DNA hybrids and G-quadruplexes at telomeres, which induces replication stress and ALT phenotypes (32,33). Prolonged loss of ATRX or DAXX also leads to progressive telomere decompaction, which gradually induces telomere dysfunction (34). However, loss of ATRX or DAXX is insufficient to cause ALT telomere elongation, implying that additional genetic or epigenetic events may cooperate with ATRX and DAXX deficiency to promote ALT development (24,31,33,35).

Orphan nuclear receptors (NRs), including COUP-TF1, COUP-TF2, TR2, TR4 and EAR2, have been shown to associate with telomeres in ALT cells (36) and are implicated in ALT development. These orphan NRs belong to the nuclear hormone receptor superfamily and NR2C/F classes and bind to telomeres via variant TCAGGG telomeric repeats, which are abundant in ALT telomeres but not in normal or telomerase-positive cells (37). Several lines of evidence support a critical role of orphan NRs in ALT regulation. COUP-TF2 and TR4 promote the telomeric localization of the zinc-finger protein 827 (ZNF827), which recruits the nucleosome remodeling and histone deacetylation (NuRD) complex to remodel telomeric chromatin structures and render telomeres more permissive for recombination (38). In addition, COUP-TF2 and TR4 can interact directly with FANCD2, a protein involved in the Fanconi anemia DNA repair pathway, to induce a DNA damage response that contributes to the ALT activity in ALT cells (39). Moreover, through bridging the interactions between ALT telomeres and their transcriptionally regulated sites, orphan NRs promote insertions of telomeric sequence throughout the genome, causing genomic instability (40).

Nevertheless, studies on the role of orphan NRs in the ALT pathway have primarily been performed on ALT cell lines, which engender confounding factors such as genetic mutations, epigenetic alterations, and telomere structural instabilities that may impair efforts to dissect the direct function of orphan NRs in ALT development. Here, we present a unique model system for ALT activation in human fibroblasts by tethering orphan NRs to telomeres. Using this system, we characterized the direct role of orphan NRs in inducing ALT phenotypes and features of ALT activity and revealed coordination between ATRX-DAXX deficiency and orphan NRs for ALT activation. Lastly, we elucidated the function of APBs in orphan NR-mediated ALT activation and the therapeutic potential of targeting APBs for ALT inhibition.

## Materials and methods

### Cell lines

Telomerase-immortalized human fibroblast BJ^T^, human osteosarcoma U2OS, and human embryonic fibroblast 293T cells were cultured in DMEM (Gibco) supplemented with 10% fetal bovine serum (FBS) and 0.5% penicillin/streptomycin. SV40-transformed WI38/VA13-2RA fibroblasts were cultured in MEM (Gibco) supplemented with 10% FBS and 0.5% penicillin/streptomycin. The Saos-2 cell line was cultured in McCoy's 5A medium supplemented with 10% FBS and 0.5% penicillin/streptomycin. All cells were grown at 37°C with 5% CO_2_.

### Plasmids

COUP-TF1-TRF1, COUP-TF2-TRF1, EAR2-TRF1, TR2-TRF1, TR4-TRF1, COUP-TF1^LBD^-TRF1, COUP-TF2^LBD^-TRF1, EAR2^LBD^-TRF1, TR2^LBD^-TRF1, TR4^LBD^-TRF1, COUP-TF1^LBD/ΔAF2^-TRF1, COUP-TF2^LBD/ΔAF2^-TRF1, COUP-TF2, TR4, COUP-TF2^ΔAF2^ and TR4^ΔAF2^ were generated using the InFusion cloning method (Clontech). Vectors were digested with BamHI and incubated overnight at 37°C (5 μg Vector, 10X CutSmart buffer 5 μl, BamHI 5 μl, H_2_O 35 μl). A QIAquick PCR purification kit was used to purify the linearized vector. Target fragments were amplified by polymerase chain reaction (PCR), and PCR products were treated with DpnI before purification. The 5X InFusion HD Enzyme Premix, linearized vector, purified PCR fragment, and dH_2_O were mixed and incubated at 50°C for 15 min. Transformation was conducted using stbl3 (ECOS™-competent cells) with ampicillin selection and overnight incubation at 37°C. Single clones were selected and amplified by liquid culture. Plasmids were collected using a QIAprep Spin Miniprep Kit and were checked by sequencing and restriction digestion. Retroviral transduction was performed to stably express the plasmids in cells. TFORF0672 (ZNF827) was a gift from Feng Zhang (Addgene plasmid #141658; http://n2t.net/addgene:141658; RRID:Addgene_141658) (41).

### RNA interference

siRNA transfections were done by reverse transfection with Lipofectamine RNAiMax (Invitrogen). All siRNAs were transfected at 25 μM as per the manufacturer's recommendations. For synchronization using thymidine and CDK1 inhibitor (CDK1i, RO-3306), cells were treated with thymidine 48 h post transfection.

### Generation of CRISPR PML-knockout cell lines

BJ^T^ cells stably expressing doxycycline-inducible Cas9 were first generated by lentiviral transduction. The stable cells were transfected with PML guide RNAs using Lipofectamine RNAiMax, according to the manufacturer's instructions (Invitrogen, USA), in parallel with doxycycline treatment (50 ng/ml) for 3 days. For U2OS and WI38-VA13/2RA PML-knockout cells, U2OS and WI38-VA13/2RA doxycycline-inducible Cas9 cells were transduced with PML guide RNAs, and then treated with doxycycline (50 ng/ml) for 3 days. Single clones were isolated using limiting dilution. Knockout efficiency was checked by immunofluorescence and western blot. Retroviral transduction was then used to stably express COUP-TF1^LBD^-TRF1, or COUP-TF2^LBD^-TRF1 in PML(+) and PML(-) BJ^T^ cells.

### Detection of telomeric DNA synthesis

To synchronize cells at G2 phase, cells were treated with 2 mM thymidine for 21 h, released into fresh medium for 4 h, and then treated with 15 mM CDK1i for 12 h. To visualize DNA synthesis, cells were incubated with 20 mM EdU for 3 h. EdU was labeled with the fluorescent dye picolyl azide via Click-it reaction (Invitrogen).

### Immunofluorescence (IF) and fluorescence *in situ* hybridization (FISH)

Cells were seeded onto coverslips inserted into 12-well plates and then fixed with 4% paraformaldehyde for 10 min and permeabilized with 0.5% Triton-X-100 (diluted in phosphate buffered saline, PBS) for 5 min. The cells were then blocked with 2% FBS (diluted in PBS) for 30 min, and incubated for 1 h each with anti-flag or anti-PML primary antibodies (1:200) and Alexa 488-conjugated secondary antibodies (1:800) diluted in 2% FBS. The cells were then fixed again with 4% paraformaldehyde for 10 min and subjected to serial dehydration in 70% EtOH, 95% EtOH and 100% EtOH for 5 min at each concentration before air-drying the coverslips. The cells were then subjected to denaturation at 80°C on a heat-plate for 3 min with hybridization mix (10 mM Tris–HCl pH 7.5, 70% formamide, 10% blocking reagent (Roche), 0.25 μM TMR-conjugated (CCCTAA)_3_ telomere PNA probe) on the slide, followed by overnight hybridization at room temperature. They were then washed twice with wash buffer A (70% formamide, 10 mM Tris-HCl pH 7.5), and then three times with wash buffer B (10 mM Tris–HCl pH 7.5, 4 M NaCl, 20% Tween-20). DAPI (Life Technology) was used to stain nuclei. Serial alcohol dehydration was performed before again air-drying the coverslips and then mounting on a slide with Prolong Gold Antifade Mountant (Life Technology).

### Chromosome orientation fluorescence *in situ* hybridization (CO-FISH)

Cells were labeled with 10 μM 3:1 BrdU: BrdC for 16 hr and then treated with 0.2 μg/ml colcemid for 4 h prior to harvesting. Cells were trypsinized and resuspended with 75 mM KCl prior to overnight fixation in 3:1 methanol/acetic acid. Cells were then dropped onto glass slides and air-dried overnight. To degrade the newly synthesized stands, slides were first rehydrated with PBS for 5 min, treated with 0.5 mg/ml RNase A for 10 min at 30°C, and labeled with 0.5 μg/ml Hoechst in 2X SSC for 15 min at RT. The slides were then exposed to long wave (365 nm) UV light for 1 h to generate DNA nicks and these were digested with 10 U/μl of Exonuclease III (Promega) for 30 min at 37°C. Slides were washed with PBS and subjected to serial dehydration in 70% EtOH, 95% EtOH and 100% EtOH for 5 min at each concentration before air-drying. For hybridization, slides were first hybridized with FAM-00-[TTAGGG]_3_ PNA probe in hybridization mix (10 mM Tris–HCl pH 7.5, 70% formamide, 10% blocking reagent (Roche) for 2 h at RT, washed with wash buffer A (70% formamide, 10 mM Tris–HCl pH 7.5), then hybridized again with TMR-00-[CCCTAA]_3_ in hybridization mix for 2 h at RT. Slides were then washed and mounted following the protocol described for FISH.

### C-circle assay

Cellular DNA was extracted using the Wizard Genomic DNA Purification Kit (Promega) and was digested using RsaI and HinfI restriction enzymes (New England BioLabs). C-circle amplifications were then performed by incubating a 40-μl reaction mixture containing 100 ng digested cellular DNA, 15U phi29 DNA polymerase (Thermo), 1 × phi29 reaction buffer, and 500 μM dNTPs at 30°C for 10 h. Following amplification, products were detected by dot blotting using ^32^P-labeled telomeric probes.

### Image acquisition and quantification

Fluorescence 3D images were acquired using a GE Healthcare DeltaVision Deconvolution microscope. Images were taken with 0.2 μm spacing for a total of 5 μm. The images were analyzed using softWoRx 5.5.1 for deconvolution and ImageJ/FIJI for quantification. First, a maximum intensity projection was applied to visualize the acquired Z-stack as a 2D-image. Then, the ‘tophat’ filter was used for background subtraction. The ‘find maxima’ command was used to identify the local maxima of signal intensity in each image, wherein the results are the segmented objects identified for each telomere or each PML-NB. Finally, overlapping signals for objects in the telomere and PML-NB images were recognized as APBs. To automatically quantify telomere and APB numbers, we utilized the ImageJ Macro language (IJM), which is a scripting language built into ImageJ.

### Xenograft model

Nude mice were obtained from the National Laboratory Animal Center, Taiwan. Thirteen 6-week-old male mice were subcutaneously injected with 1 × 10^7^ SaOS2 cells suspended in serum free DMEM on either flank. The mice were randomly allocated to treatment with either As_2_O_3_ (2 mg/kg) or equivalent volume of PBS. As_2_O_3_ and PBS treatment was started 1 week after inoculation when the mice had developed palpable tumors. Both As_2_O_3_ and PBS were administered intraperitoneally for 3–5 consecutive days. This study was carried out in strict adherence with the recommendations from the IACUC (21-12-1754) of Academia Sinica, Taiwan, and efforts were made to minimize the number of animals used for the study.

### Statistical analyses

GraphPad Prism 6 and Microsoft Excel were used to generate tables and graphs. For cell-based imaging experiments, each experiment was conducted two to three times, with a minimum of 100 cells analyzed per experiment, resulting in approximately 200–300 cells analyzed per sample. In the scatter plots, each dot represents the quantified number per cell and the red lines indicate either the median or mean to facilitate comparison between samples. Statistical analyses were performed using either two-tailed Mann–Whitney *U* tests or unpaired t-tests as described in the figure legends. For mouse-based imaging experiments, a total of seven tumors from six PBS-treated mice were compared to eight tumors from seven As_2_O_3_-treated mice. A minimum of 200 cells were analyzed for each tumor. In the graphs, each dot indicates the average number quantified per cell within one tumor. Statistical analyses were performed using unpaired *t*-tests.

## Results

### Tethering orphan NRs to telomeres triggers ALT induction in human fibroblasts

**Figure 1.**
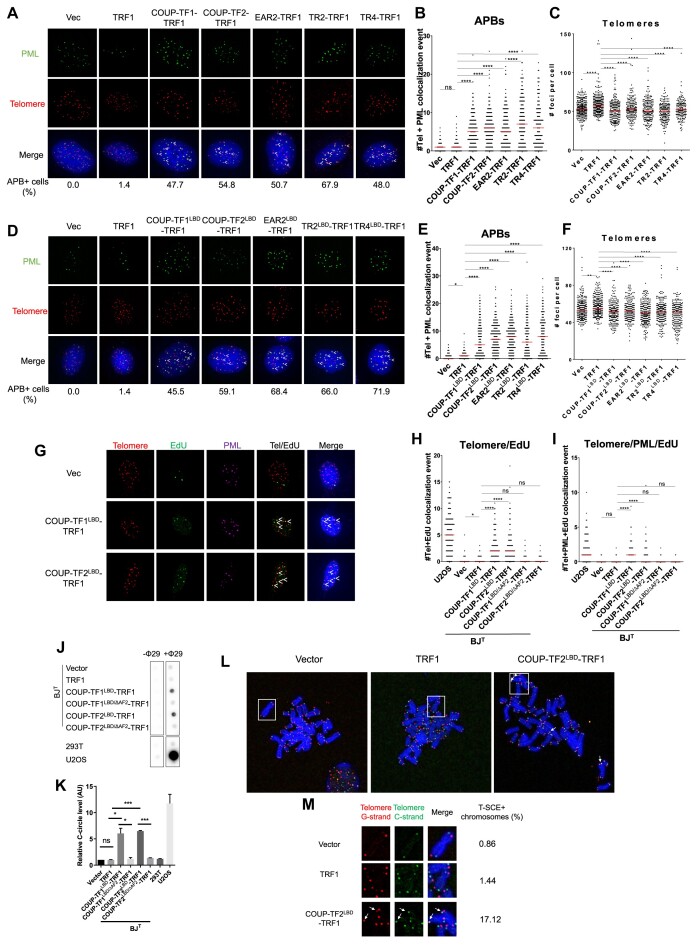
Tethering of orphan NRs to telomeres triggers ALT induction in human fibroblasts. (**A**) Representative images showing PML and telomere co-localization in BJ^T^ cells expressing the full-length orphan NRs COUP-TF1, COUP-TF2, TR2, TR4 or EAR-2. PML was detected by IF, and telomeres were detected by FISH using the TelC PNA probe. Co-localization of PML (green) and telomeres (red) appears yellow. White arrows indicate APBs. ‘APB + cells (%)’ values reflect that more than five telomere + PML co-localization events were observed in a given cell. Quantification of APBs (**B**) and telomere numbers (**C**) in individual BJ^T^ cells (n > 100) expressing the full-length orphan NRs COUP-TF1, COUP-TF2, TR2, TR4 or EAR2. (**D**) Representative images showing co-localization of PML and telomeres in BJ^T^ cells expressing the LBD of orphan NRs COUP-TF1, COUP-TF2, TR2, TR4 or EAR2. Quantification of APBs (**E**) and telomere numbers (**F**) in individual BJ^T^ cells (*n* > 100) expressing LBDs of orphan NRs COUP-TF1, COUP-TF2, TR2, TR4 or EAR2. (**G**) Representative images showing EdU at telomeres and PML in BJ^T^-COUP-TF1^LBD^-TRF1 and BJ^T^-COUP-TF2^LBD^-TRF1 cells. EdU and PML were detected by IF, and telomeres were detected by FISH using the TelC PNA probe. Co-localization of EdU (green), PML (magenta), and telomeres (red) appears white. White arrows indicate telomeric DNA synthesis at APBs. Quantification of telomere and EdU co-localization (**H**) and telomere, PML and EdU co-localization (**I**) in BJ^T^-COUP-TF1^LBD^-TRF1 and BJ^T^-COUP-TF2^LBD^-TRF1 cells (*n* > 100). (**J**) C-circles detected in BJ^T^-COUP-TF1^LBD^-TRF1 and BJ^T^-COUP-TF2^LBD^-TRF1 cells. (**K**) Quantification of C-circles detected in (J). (**L**) Representative images showing T-SCEs in BJ^T^-COUP-TF2^LBD^-TRF1 cells. Co-localization of TelG (green) and TelC (red) appears yellow. White arrows indicate T-SCEs. (**M**) Zoom-in view of the boxed region in (L) and quantification of T-SCEs detected in (L) from two independent experiments (*n* > 500). Red lines represent median of two independent experiments. ns *P*> 0.05, *P< 0.05, **P< 0.01, ***P< 0.001, ****P< 0.0001, as determined by Mann–Whitney *U* test.

We established an experimental model to recapitulate telomeric recruitment of orphan NRs in primary fibroblast cells immortalized with telomerase (BJ^T^) for the investigation of how orphan NRs contribute to ALT activation. The orphan NRs COUP-TF1, COUP-TF2, TR2, TR4 and EAR2 were tethered to telomeres in BJ^T^ cells through ectopic stable expression as a fusion protein with the telomere binding protein TRF1. We visualized the cellular localization of the orphan NR-TRF1 fusion proteins by immunofluorescence (IF) staining. Our IF data revealed orphan NR nuclear foci co-localized with telomeric signals from fluorescence *in situ* hybridization (FISH), indicating the telomeric localization of orphan NR-TRF1 fusion proteins (Supplementary Figure S1A). Next, we investigated the ability of orphan NRs to induce ALT phenotypes upon their targeting to telomeres in BJ^T^ cells. Using IF with antibodies against PML in combination with telomere FISH, we observed robust co-localization between PML-NBs and telomeres in BJ^T^ cells expressing COUP-TF1-TRF1, COUP-TF2-TRF1, TR2-TRF1, TR4-TRF1 or EAR2-TRF1 relative to vector control cells or cells overexpressing TRF1 (Figure 1A,B), suggesting that targeting orphan NRs to telomeres induces APB formation. Using an established protocol for quantifying telomere FISH signal (42), we assessed telomere clustering upon telomeric targeting of orphan NRs. Our results show that expression of orphan NR-TRF1 fusion proteins in BJ^T^ cells caused a decrease in the number of telomere foci in comparison to cells overexpressing TRF1, indicating that telomere targeting of orphan NRs induces telomere clustering (Figure 1C).

The C-terminal ligand binding domain (LBD) of orphan NRs has been shown to be critical for their transcriptional activity (43). Therefore, we coupled the LBDs of COUP-TF1, COUP-TF2, TR2, TR4 and EAR2 with TRF1 and generated BJ^T^ cells expressing orphan NR LBD-TRF1 fusion proteins (Supplementary Figure S1B). We also found that upon targeting to telomeres, the LBDs of orphan NRs is sufficient to induce APB formation (Figure 1D,E) and telomere clustering, evidenced by a decrease in telomere number (Figure 1F) and an increase in telomere volume (Supplementary Figure S2A). This ALT induction by LBDs of orphan NRs is often to a higher degree than that detected for the full-length proteins. This is presumably due to the specific association of LBD-TRF1 proteins with telomeres, whereas full-length orphan NR-TRF1 proteins may engage in both telomere and non-telomere bindings.

We have shown above that telomere tethering of orphan NRs in human fibroblast cells induces ALT phenotypes, including APB formation and telomere clustering, so we were interested in investigating if this then induces ALT activity. To examine that possibility, we investigated the induction of other ALT phenotypes, such as ALT telomere DNA synthesis, telomeric C-circle generation, and telomere sister chromatid exchange (T-SCEs), which are features of ALT activity associated with telomere recombination and elongation (44). Specifically, we determined levels of recombination-based ALT telomere DNA synthesis during the G2 phase of the cell cycle in BJ^T^ cells upon orphan NR recruitment. The cells were synchronized in G2 phase by means of thymidine and CDK1 inhibitor treatments, followed by addition of EdU (5-ethynyl-2′-deoxyuridine) to detect DNA synthesis (7). Upon conducting Click chemistry for EdU visualization and telomeric FISH, co-localizations between telomeres and EdU foci were identified in BJ^T^ cells expressing COUP-TF1^LBD^-TRF1 and COUP-TF2^LBD^-TRF1, indicative of non-S phase DNA synthesis at telomeres (Figure 1G,H). Moreover, EdU-associated telomeres also co-localized with PML foci, indicating telomere synthesis at APBs (Figure 1G,I). Thus, our data indicate that targeting orphan NRs to telomeres induces ALT telomere DNA synthesis at APBs in human fibroblasts. Moreover, accumulation of extra chromosomal telomeric C-circles as recombination byproducts has been shown to be associated with ALT telomere recombination (45). Accordingly, we extracted genomic DNA from control BJ^T^ cells, as well as TRF1, COUP-TF1^LBD^-TRF1 and COUP-TF2^LBD^-TRF1-expressing BJ^T^ cells, and used it for C-circle assay. Our results show that telomeric targeting of the LBDs of COUP-TF1 or COUP-TF2 induced C-circle accumulation in BJ^T^ cells (Figure 1J,K). Additionally, T-SCEs are direct evidence of ALT recombination (46). We conducted chromosome orientation FISH (CO-FISH) and found that tethering COUP-TF2^LBD^ to telomeres of BJ^T^ cells induces T-SCEs (Figure 1L,M). Thus, together, our data indicate that telomeric recruitment of orphan NRs induces features of ALT activity, suggesting ALT induction.

To demonstrate the reproducibility of our ALT-inducing model, we also targeted the COUP-TF2^LBD^ to the telomeres of three additional primary fibroblast cells—BJ, IMR90 and WI38 (Supplementary Figure S1C). We observed ALT induction evidenced by APB formation (Supplementary Figure S2B–D), telomere clustering (Supplementary Figure S2E), and telomere DNA synthesis (Supplementary Figure S2F–G) upon expression of COUP-TF2^LBD^-TRF1 in BJ, IMR90 and WI38 cells. ALT induction analysis in the additional fibroblasts revealed consistent results with BJ^T^ cells, validating that telomeric targeting of orphan NRs induces ALT in human fibroblasts. By performing IF against shelterin proteins TRF2 and RAP1 with telomere FISH, we found that telomeric targeting of COUP-TF2^LBD^ by TRF1 does not prevent shelterin localization to telomeres (Supplementary Figure S1D). Upon investigating for DNA damage marked by co-localization of phosphorylated histone H2AX (γH2AX) and telomeres or telomere dysfunction-induced foci (TIFs), which has been previously shown to be elevated upon ALT induction (11–14,37), we did not observe an increase of DNA damage response (DDR) upon tethering of orphan NRs to telomeres of fibroblasts (Supplementary Figure S3A–D). This discovery suggests our innovative model as a unique mechanism independent of telomere DDR for ALT induction.

### Orphan NRs mediate APB formation and ALT telomere DNA synthesis in ALT cells

**Figure 2.**
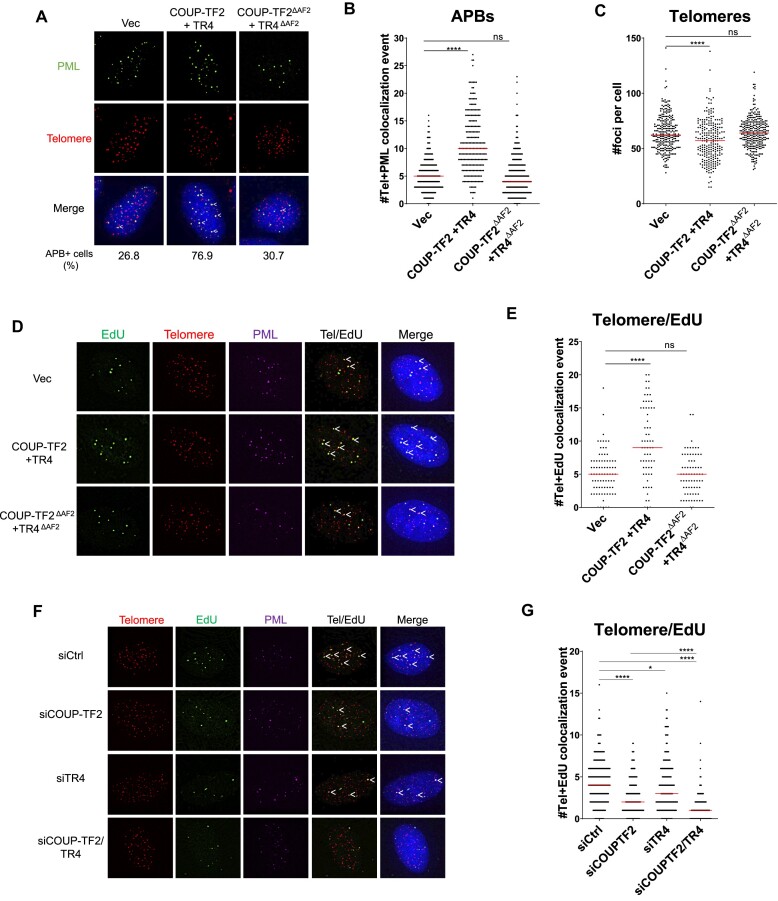
Orphan NRs mediate APB formation and ALT telomere DNA synthesis in ALT cells. (**A**) Representative images showing PML and telomere co-localization in U2OS cells overexpressing COUP-TF2 and TR4. Quantification of APBs (**B**) and telomere numbers (**C**) in individual U2OS cells (*n* > 100) expressing orphan NRs COUP-TF2 and TR4 or COUP-TF2^ΔAF2^ and TR4^ΔAF2^. (**D**) Representative images showing EdU at telomeres and PML in U2OS-COUP-TF2 + TR4 cells. EdU and PML were detected by IF, and telomeres were detected by FISH using the TelC PNA probe. Co-localization of EdU (green), PML (magenta), and telomeres (red) appears white. White arrows indicate telomeric DNA synthesis at APBs. (**E**) Quantification of telomere and EdU co-localization in U2OS-COUP-TF2 + TR4 cells. (**F**) Representative images showing reduced EdU signal at telomeres and PML levels in U2OS cells upon treatment with siRNAs against COUP-TF2 or TR4. (**G**) Quantification of telomere and EdU co-localization in U2OS cells upon treatment with siRNAs against COUP-TF2 or TR4. Cells were synchronized in G2 phase by thymidine and CDK1i treatments for 21 h and 12 h, respectively. Red lines represent median of two independent experiments. ns *P*> 0.05, *P< 0.05, **P< 0.01, ***P< 0.001, ****P< 0.0001, as determined by Mann–Whitney *U* test.

We have shown that targeting orphan NRs to telomeres in human fibroblasts induces APB formation, telomere clustering, C-circle generation, and telomeric DNA synthesis at APBs. To determine if all these roles of orphan NRs are consistent in ALT cells, we overexpressed COUP-TF2 and TR4 in U2OS and WI38-VA13/2RA ALT cell lines, which exhibit pre-existing ALT phenotypes and activity. Telomeric localization of ectopically expressed COUP-TF2 and TR4 proteins was observed (Supplementary Figure S1E), which is consistent with the presence of abundant TCAGGG repeat sequences in ALT telomeres (37). Furthermore, co-expression of COUP-TF2 and TR4 significantly enhanced APB formation (Figure 2A, B) and telomere DNA synthesis (Figure 2D,E), while only minimally increased telomere clustering (Figure 2C), in U2OS cells. In WI38-VA13/2RA cells, simultaneous overexpression of COUP-TF2 and TR4 resulted in a marginal increase of APB formation (Supplementary Figure S4A), but did not promote further telomere clustering (Supplementary Figure S4B) and telomere DNA synthesis (Supplementary Figure S4C), presumably due to pre-existing high-level of ALT phenotypes and telomeric association of endogenous orphan NRs. Our overexpression experiments indicate that orphan NRs may promote APB formation and telomere DNA synthesis when they are localized to telomeres in ALT cells.

To delineate the contribution of specific orphan NRs, particularly COUP-TF2 and TR4, in promoting ALT, we depleted COUP-TF2 and/or TR4 from ALT cell lines by siRNAs (Supplementary Figure S4D) and then subjected them to ALT induction analyses. In U2OS cells, we observed that depletion of COUP-TF2 reduced APB formation (Supplementary Figure S4E) and telomeric DNA synthesis (Figure 2F, G), but not telomere clustering (Supplementary Figure S4F). However, depletion of TR4 did not lead to a decrease in APB formation (Supplementary Figure S4E) and telomere clustering (Supplementary Figure S4F) but it slightly reduced telomere DNA synthesis (Figure 2F, G). Upon co-depletion of COUP-TF2 and TR4, a further reduction in telomere DNA synthesis was observed (Figure 2F, G). These findings suggest that COUP-TF2 contributes to ALT activation in U2OS cells more significantly than TR4. In WI38-VA13/2RA cells, similar to what was identified in U2OS cells, COUP-TF2 depletion resulted in significant reductions of APB formation (Supplementary Figure S4G) and telomere DNA synthesis (Supplementary Figure S4H) but not telomere clustering (Supplementary Figure S4I). These knockdown experiments highlight the role of orphan NRs, particularly COUP-TF2, in promoting APB formation and telomere DNA synthesis in U2OS and WI38-VA13/2RA cells. However, our findings suggest that orphan NRs may not play a major role in telomere clustering in these ALT cells, which differs from our observation in fibroblasts (Figure 1C, F). Our findings also reveal that specific orphan NRs may have different and/or compensatory roles in promoting ALT activation.

### The AF2 domain of orphan NRs and ZNF827 are critical for ALT induction

**Figure 3.**
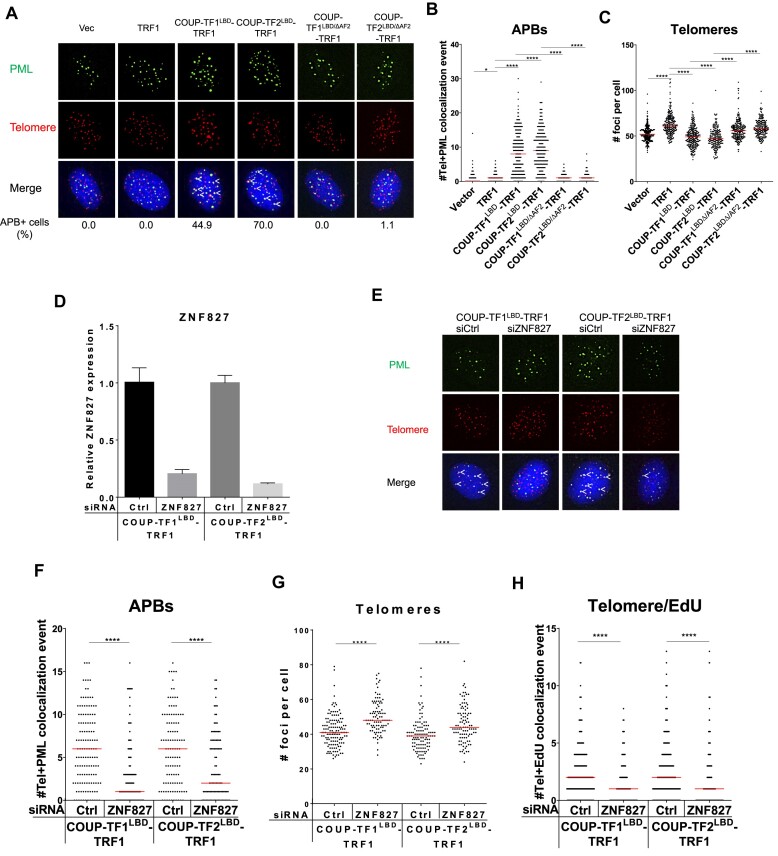
The AF2 domain of orphan NRs is critical for ALT induction. (**A**) Representative images showing loss of APBs in BJ^T^-COUP-TF1^LBD/ΔAF2^-TRF1 and BJ^T^-COUP-TF2^LBD/ΔAF2^-TRF1 cells. PML was detected by IF, and telomeres were detected by FISH using the TelC PNA probe. Co-localization of PML (green) and telomeres (red) appears yellow. White arrows indicate APBs. Quantification of APBs (**B**) and telomere numbers (**C**) in individual BJ^T^ cells (n > 100) expressing COUP-TF1^LBD^-TRF1, COUP-TF2^LBD^-TRF1, COUP-TF1^LBD/ΔAF2^-TRF1 or COUP-TF2^LBD/ΔAF2^-TRF1. (**D**) Relative ZNF827 expression in BJ^T^ cells expressing COUP-TF1^LBD^-TRF1 or COUP-TF2^LBD^-TRF1, as measured by qPCR. (**E**) Representative images showing reduced APB formation in BJ^T^-COUP-TF1^LBD^TRF1 and BJ^T^-COUP-TF2^LBD^TRF1 cells upon treatment with siRNAs against ZNF827. Quantification of APBs (**F**), telomere numbers (**G**), and telomere and EdU co-localization (**H**) in individual BJ^T^ cells (*n* > 100) expressing COUP-TF1^LBD^-TRF1 or COUP-TF2^LBD^-TRF1 upon treatment with siRNAs against ZNF827. Red lines represent median of two independent experiments. ns *P*> 0.05, *P< 0.05, **P< 0.01, ***P< 0.001, ****P< 0.0001, as determined by Mann–Whitney *U* test.

Next, we investigated how orphan NRs promote ALT activation. The LBD of orphan NRs contains the transcriptional function AF2 domain that is responsible for recruiting transcriptional co-regulators (43). We reasoned that the AF2 domain might mediate the ALT induction triggered by telomeric targeting of orphan NRs. To examine this possibility, the AF2 domain at the C-terminus of the LBDs of COUP-TF1 and COUP-TF2 were deleted to generate BJ^T^ cells expressing COUP-TF1^LBD/ΔAF2^-TRF1 and COUP-TF2^LBD/ΔAF2^-TRF1 (Supplementary Figure S1F). We found that removing the AF2 domain from COUP-TF1^LBD^ and COUP-TF1^LBD^ prevented APB formation (Figure 3A,B), telomere clustering (Figure 3C), C-circle formation (Figure 1J, K), and telomere DNA synthesis (Figure 1H, I) upon their telomeric targeting through TRF1 fusion in BJ^T^ cells. Similarly, ectopic expression of COUP-TF2^ΔAF2^ and TR4^ΔAF2^ in U2OS and WI38-VA13/2RA cells failed to induce features of ALT activity (Figure 2A-E, Supplementary Figure S4A–C). These data support that the AF2 domain of orphan NRs is critical for their ability to induce ALT upon telomere recruitment.

Moreover, previous studies have shown that APB formation and ALT activity are regulated by cellular processes such as chromatin remodeling and sumoylation (15,38). Therefore, we reasoned that depleting proteins involved in these processes from BJ^T^ cells expressing COUP-TF1^LBD^-TRF1 and COUP-TF2^LBD^-TRF1 might perturb ALT induction. It has been shown previously that the zinc finger protein ZNF827 is localized at the telomeres of ALT cells, where it promotes APB formation and recruits the nucleosome remodeling and histone deacetylation (NuRD) complex to facilitate homologous recombination (38). We used siRNAs to deplete ZNF827 from BJ^T^-COUP-TF1^LBD^-TRF1 and BJ^T^-COUP-TF2^LBD^-TRF1 cells (Figure 3D) and then assessed ALT induction. We found that ZNF827 depletion significantly reduced APB formation (Figure 3E,F), increased telomere number (Figure 3G), and decreased telomere DNA synthesis (Figure 3H). In addition, overexpression of ZNF827 in BJ^T^-COUP-TF2^LBD^-TRF1 cells enhanced APB formation but its overexpression in BJ^T^-COUP-TF2^LBD/ΔAF2^-TRF1 could not rescue the ALT phenotype (Supplementary Figure S5A). These findings indicate that the ability of orphan NRs to induce ALT activation in BJ^T^ cells is dependent on ZNF827. Moreover, it has been shown previously that the Fanconi anemia protein FANCD2 interacts with COUP-TF2 and TR4 to promote ALT activity in ALT cells (39). Using siRNAs to deplete FANCD2 from BJ^T^-COUP-TF2^LBD^-TRF1 cells, we observed that loss of FANCD2 expression did not affect APB formation (Supplementary Figure S5B), telomere clustering (Supplementary Figure S5C), or telomere DNA synthesis (Supplementary Figure S5D). In addition, the MMS21-SMC5-SMC6 SUMO E3 ligase complex has been shown to promote APB formation in ALT cells by sumoylating TRF1 and TRF2 (15). Using IF staining, we found that the recombination proteins SMC5, SMC6 and NBS1 localized at PML-NBs in parental BJ^T^ fibroblasts (Supplementary Figure S5E), and were further identified at telomeres upon expressing COUP-TF1^LBD^-TRF1 or COUP-TF2^LBD^-TRF1 (Supplementary Figure S5F-H), suggesting that APB formation results in their telomeric localization. However, depletion of NBS1, SMC5 or SMC6 by siRNA transfection (Supplementary Figure S5I) in BJ^T^-COUP-TF1^LBD^-TRF1 and BJ^T^-COUP-TF2^LBD^-TRF1 cells did not prevent either APB formation (Supplementary Figure S5J) or telomere clustering (Supplementary Figure S5K), indicating that these recombination and repair proteins may not be involved in orphan NR-mediated ALT activation. Thus, these findings suggest that orphan NRs induce ALT activation in primary fibroblasts via their AF2 domains and this activity is dependent on ZNF827.

### ATRX/DAXX depletion synergizes with telomeric recruitment of orphan NRs for ALT activation

**Figure 4.**
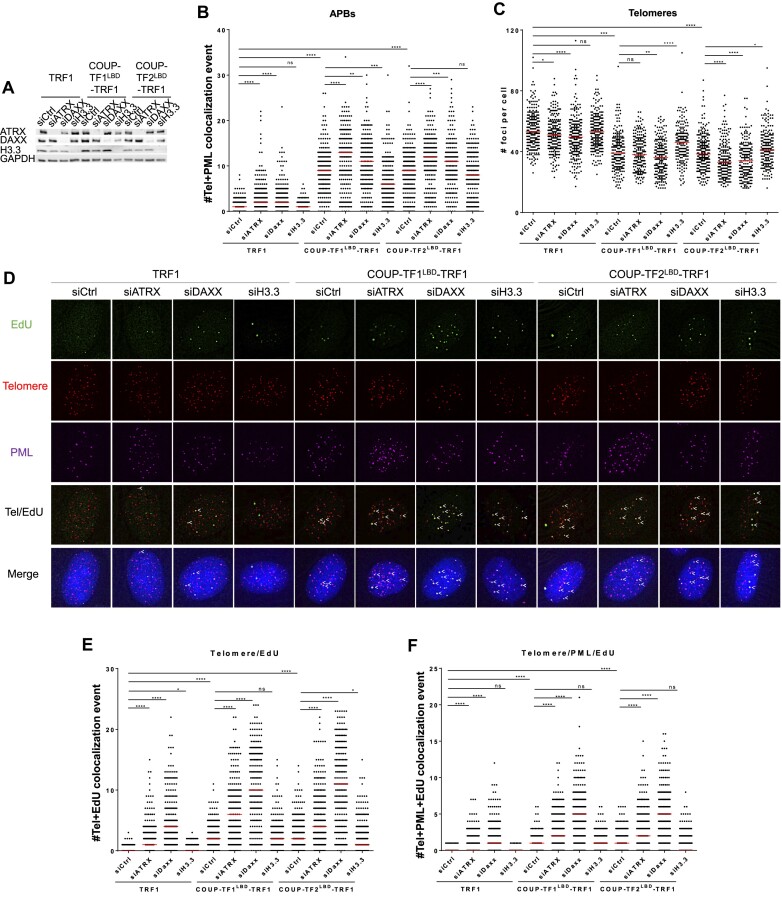
ATRX/DAXX depletion combined with orphan NR recruitment to telomeres promotes features of ALT activity more dramatically. (**A**) Western blot showing the loss of protein expression in BJ^T^-COUP-TF1^LBD^-TRF1 and BJ^T^-COUP-TF2^LBD^-TRF1 cells upon treatment with siRNAs against ATRX, DAXX or H3.3. Quantification of APBs (**B**) and telomere numbers (**C**) in BJ^T^-COUP-TF1^LBD^-TRF1 and BJ^T^-COUP-TF2^LBD^-TRF1 cells (*n* > 100) upon treatment with siRNAs against ATRX, DAXX, or H3.3. (**D**) Representative images showing EdU at telomeres and PML in BJ^T^-COUP-TF1^LBD^-TRF1 and BJ^T^-COUP-TF2^LBD^-TRF1 cells upon treatment with siRNAs against ATRX, DAXX or H3.3. EdU and PML were detected by IF, and telomeres were detected by FISH using the TelC PNA probe. Co-localization of EdU (green), PML (magenta) and telomeres (red) appears white. White arrows indicate telomeric DNA synthesis at APBs. Quantification of telomere and EdU co-localization (**E**) and of telomere, PML and EdU co-localization (**F**) in BJ^T^-COUP-TF1^LBD^-TRF1 and BJ^T^-COUP-TF2^LBD^-TRF1 cells (*n* > 100) upon treatment with siRNAs against ATRX, DAXX or H3.3. Cells were synchronized in G2 phase by means of thymidine and CDK1i treatments for 21 h and 12 h, respectively. Red lines represent median of two independent experiments. ns *P*> 0.05, *P< 0.05, **P< 0.01, ***P< 0.001, ****P< 0.0001, as determined by Mann–Whitney *U* test.

The ALT suppressors ATRX and DAXX form a histone chaperone complex that deposits histone variant H3.3 on telomeres (23,24,27,28,30). It has been shown previously that loss of ATRX or DAXX may promote ALT phenotypes and ALT activation (34). As described above, we found that tethering orphan NRs to telomeres in BJ^T^ fibroblast cells induced ALT induction. Therefore, we wondered if these two ALT events, i.e. orphan NR recruitment to telomeres and loss of ATRX or DAXX, can coordinate to promote ALT activation in BJ^T^ cells. To examine this possibility, we depleted ATRX, DAXX, as well as histone H3.3 expression by siRNA transfection in BJ^T^ cells and then conducted ALT induction analyses (Figure 4A). We observed that depletion of ATRX or DAXX from BJ^T^ cells elicited low-level APB formation (Figure 4B). Moreover, in BJ^T^-COUP-TF1^LBD^-TRF1 and BJ^T^-COUP-TF2^LBD^-TRF1 cells, we detected a further increase in APBs and telomere clustering upon ATRX or DAXX depletion (Figure 4B, C). Furthermore, depletion of ATRX or DAXX triggered telomeric DNA synthesis at APBs (Figure 4D–F), which was further enhanced when combined with expression of COUP-TF1^LBD^-TRF1 or COUP-TF2^LBD^-TRF1 (Figure 4D–F). It is worth noting that depletion of histone H3.3 did not promote ALT induction in BJ^T^ fibroblasts or in BJ^T^-COUP-TF1^LBD^-TRF1 or BJ^T^-COUP-TF2^LBD^-TRF1 cells. These combinatorial analyses indicate that orphan NRs may cooperate with ATRX and DAXX deficiencies to promote ALT activation, independently of histone H3.3 deposition.

### APB is critical for orphan NR-induced telomeric DNA synthesis

**Figure 5.**
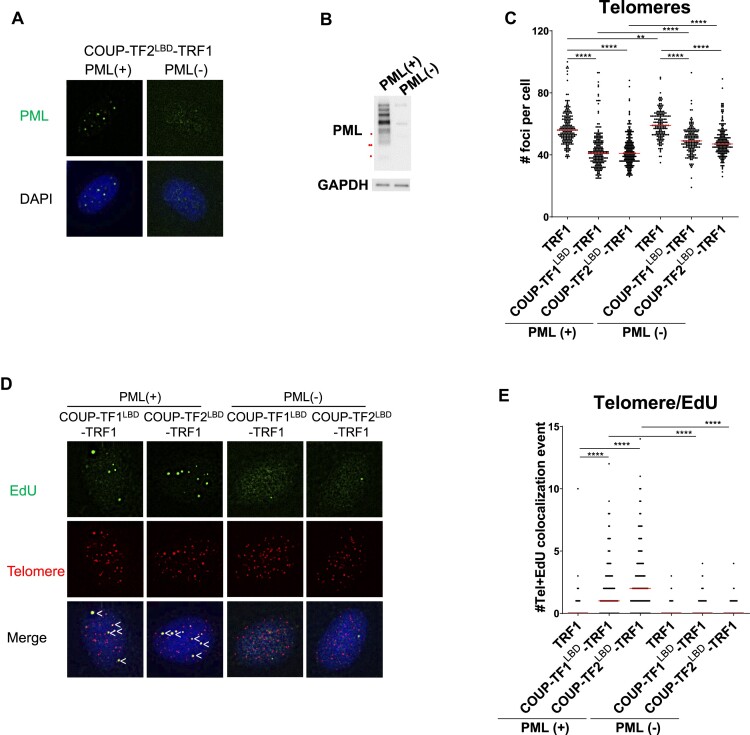
PML is critical for orphan NR-induced telomeric DNA synthesis at APBs. (**A**) Representative images of PML(+) and PML(–) BJ^T^-COUP-TF2^LBD^-TRF1 cells. PML was detected by IF. (**B**) Western blot showing PML protein expression in PML(+) and PML(–) BJ^T^ cells. (**C**) Quantification of telomere number in PML(+) and PML(–) BJ^T^ cells (*n* > 100). (**D**) Representative images showing EdU at telomeres in PML(+) and PML(–) BJ^T^ cells. EdU were detected by IF, and telomeres were detected by FISH using the TelC PNA probe. Co-localization of EdU (green) and telomeres (red) appears yellow. White arrows indicate telomeric DNA synthesis. (**E**) Quantification of telomere and EdU co-localization in PML(+) and PML(–) BJ^T^ cells (*n* > 100). Cells were synchronized in G2 phase by means of thymidine and CDK1i treatments for 21 h and 12 h, respectively. Red lines represent median of two independent experiments. ns *P*> 0.05, *P< 0.05, **P< 0.01, ***P< 0.001, ****P< 0.0001, as determined by Mann–Whitney *U* test.

We have shown that tethering orphan NRs to telomeres induces features of ALT activity in BJ^T^ cells. To investigate the significance of APB formation for ALT induction, we generated PML knockout BJ^T^ cells by means of CRISPR/Cas9 technology using guide RNAs targeting exons 1–3 of the *PML* gene for expressing COUP-TF1^LBD^-TRF1 and COUP-TF2^LBD^-TRF1. Our IF staining (Figure 5A) and Western blotting (Figure 5B) analyses showed that the knockout cells lacked expression of PML isoforms, indicating loss of APBs. Upon expression of COUP-TF1^LBD^-TRF1 or COUP-TF2^LBD^-TRF1, telomere clustering partially decreased in the BJ^T^ PML knockout cells (Figure 5C), in agreement with previous reports that APB formation promotes telomere clustering (18,47). Interestingly, substantial telomere clustering still occurred in the BJ^T^ PML knockout cells expressing COUP-TF1^LBD^-TRF1 or COUP-TF2^LBD^-TRF1 (Figure 5C), suggesting that there may be an APB-independent mechanism for telomere clustering. Furthermore, we investigated if the telomere DNA synthesis induced by orphan NRs requires PML. Intriguingly, telomere DNA synthesis was not observed in PML knockout BJ^T^ cells expressing COUP-TF1^LBD^-TRF1 or COUP-TF2^LBD^-TRF1 (Figure 5D,E), suggesting that APB is critical for ALT activation induced by recruitment of orphan NRs to telomeres. To investigate if PML is required for ALT telomere DNA synthesis in ALT cells, we also generated PML knockout U2OS and WI38-VA13/2RA cells (Supplementary Figure S6A) and again observed that telomere clustering was unaffected (Supplementary Figure S6B,C) and telomeric DNA synthesis was abolished (Supplementary Figure S6D,E). These results are consistent with our experiments on BJ^T^ cells and support findings by others (7,19) that PML is critical for telomeric DNA synthesis in ALT cells.

### Arsenic trioxide disrupts APBs and inhibits ALT telomere DNA synthesis in ALT cells

As described above, APBs are critical to orphan NR-mediated ALT induction, so we hypothesized that drugs targeting PML for degradation could also inhibit ALT activity. Arsenic trioxide (As_2_O_3_) is a therapeutic drug used for the treatment of acute promyelocytic leukemia (APL) by promoting degradation of the PML–retinoic acid receptor-α (PML-RAR) oncogene (48). It can also bind to PML and promote its proteolysis (49,50). Aside from its use in APL, As_2_O_3_ has been demonstrated to kill various types of cancer cells, such as liver cancer, lung cancer, breast cancer, and glioma, among others, and thus also has been subjected to clinical trials for these multiple cancer types (51). As_2_O_3_ has also been reported to inhibit cell proliferation and induce cell death in osteosarcoma cell lines, including U2OS and SaOS2 ALT cells (52), but whether As_2_O_3_ has the ability to inhibit ALT in ALT cells has not been explored.

**Figure 6.**
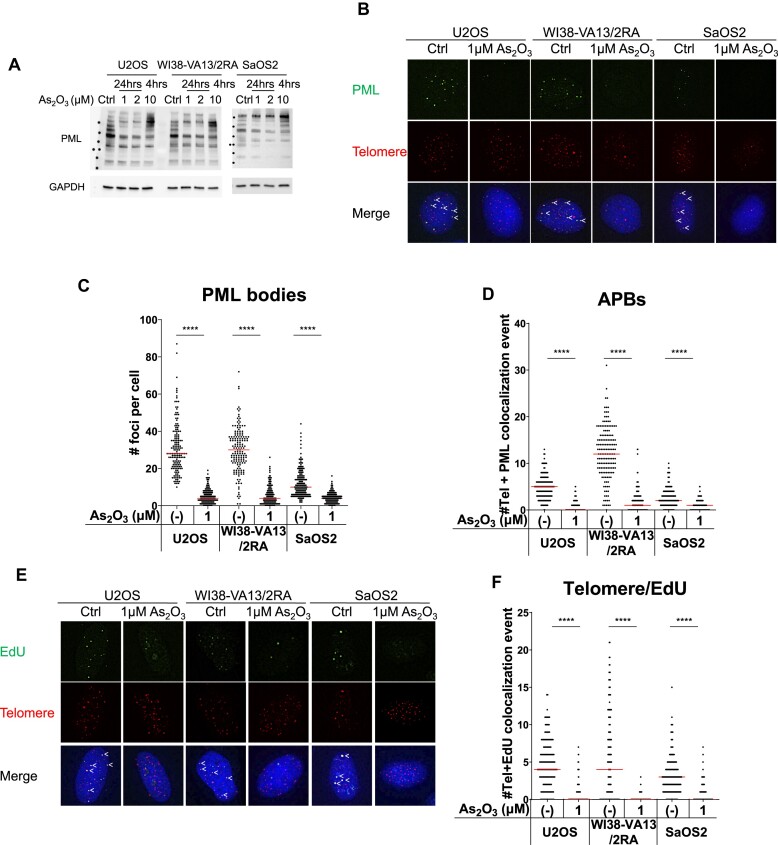
Arsenic trioxide disrupts APBs and inhibits ALT telomere DNA synthesis in ALT cells. (**A**) Western blot showing loss of PML isoforms upon 1 μM or 2 μM As_2_O_3_ treatment of U2OS, WI38-VA13/2RA, and SaOS2 cells. (**B**) Representative images showing loss of PML and telomere + PML co-localization in U2OS, WI38-VA13/2RA and SaOS2 cells upon As_2_O_3_ treatment. PML was detected by IF, and telomeres were detected by FISH using the TelC PNA probe. Co-localization of PML (green) and telomeres (red) appears yellow. White arrows indicate APBs. Quantification of PML bodies (**C**) and APBs (**D**) in individual U2OS (*n* > 100), WI38-VA13/2RA (*n* > 100), and SaOS2 (*n* > 100) cells. (**E**) Representative images showing loss of EdU at telomeres in U2OS, WI38-VA13/2RA and SaOS2 cells upon As_2_O_3_ treatment. EdU were detected by IF and telomeres were detected by FISH using the TelC PNA probe. Co-localization of EdU (green) and telomeres (red) appears yellow. White arrows indicate telomeric DNA synthesis. (**F**) Quantification of telomere and EdU co-localization in individual U2OS (*n* > 100), WI38-VA13/2RA (*n* > 100) and SaOS2 (*n* > 100). Red lines indicate median of two independent experiments. ns *P*> 0.05, *P< 0.05, **P< 0.01, ***P< 0.001, ****P< 0.0001, as determined by Mann–Whitney *U* test.

Thus, we sought to investigate the possibility of targeting ALT using As_2_O_3._ As_2_O_3_ is a poison with profound cell cytotoxicity through inducing oxidative stress and cell apoptosis (50,53). We reasoned that severe cell death could prevent accurate measurements of ALT phenotypes. Therefore, we treated cells with various concentrations of As_2_O_3_ over a 48-h period and observed cell growth, in order to identify optimal conditions for the experiments. Consistent with its general hypertoxicity, we observed that As_2_O_3_ at 10 μM elicited a robust cell death of U2OS, WI38-VA13/2RA and SaOS2 ALT cells within 24 h (Supplementary Figure S7A). Although a shorter treatment duration of 4 h at 10 μM allowed for improved cell survival (Supplementary Figure S7A), this was not sufficient to degrade PML isoforms (Figure 6A). At lower concentrations, we observed that significant cell death still occurred upon treatments of 1 μM As_2_O_3_ over 48 hours or 2 μM over 24 h, whereas cells could tolerate 1 μM of As_2_O_3_ for 24 h (Supplementary Figure S7A). Moreover, Western blotting and IF-telomere FISH revealed that treatment with 1 μM of As_2_O_3_ for 24 h was sufficient to degrade PML proteins (Figure 6A) and to disrupt PML bodies in U2OS, WI38-VA13/2RA and SaOS2 ALT cells (Figure 6B, C), resulting in loss of APBs in these ALT cells (Figure 6B, D). Following this loss of APBs, we also observed that As_2_O_3_ treatment prevented telomere DNA synthesis (Figure 6E, F). Thus, our *in vitro* analysis indicates that As_2_O_3_ may inhibit ALT activity by disrupting APB formation in ALT cells.

### Arsenic trioxide suppresses APB formation and features of ALT activity in SaOS2 xenografts in mice

**Figure 7.**
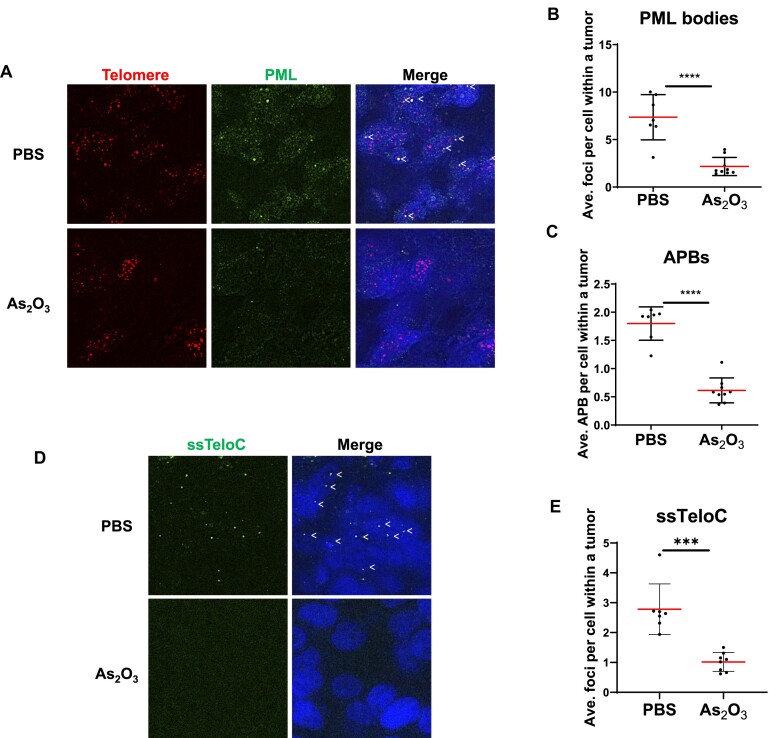
Arsenic trioxide suppresses APB formation and features of ALT activity in SaOS2 xenografts in mice. (**A**) Representative images APBs in mouse SaOS2 xenografts. ALT osteosarcoma cell lines were injected into nude mice for tumor formation and then 6 mice were treated with PBS as control (7 tumors) and 7 mice were treated with As_2_O_3_ (8 tumors). PML was detected by IF, and telomeres were detected by FISH using the TelC PNA probe. Co-localization of PML (green) and telomeres (red) appears yellow. White arrows indicate APBs. Loss of PML bodies (**B**) and telomere + PML co-localization (**C**) in mouse xenografts treated with As_2_O_3_. (**D**) Representative images of single-stranded C-rich telomeric DNA (ssTeloC) detected in mouse SaOS2 xenografts. ssTeloC signals were detected by native FISH using the TelG PNA probe (**E**) Loss of ssTeloC or ALT activity detected by the native FISH assay in mouse xenografts treated with As_2_O_3_. Red lines indicate the mean. ns *P*> 0.05, *P< 0.05, **P< 0.01, ***P< 0.001, ****P< 0.0001, as determined by unpaired *t*-test.

To investigate the therapeutic potential of As_2_O_3_ on ALT inhibition, we examined the ability of As_2_O_3_ to target PML bodies and APB formation in tumor xenografts generated by subcutaneously injecting SaOS2 ALT cancer cells into nude mice. The mice carrying tumors were treated with PBS as a control or As_2_O_3_ for 3–5 consecutive days. We collected the tumors and performed PML IF staining and telomere FISH to investigate APB formation. PML bodies could be visualized in control SaOS2 tumors, and they co-localized with telomere signals, indicative of APB formation in SaOS2 mouse xenografts (Figure 7A–C). However, following As_2_O_3_ treatment, we detected a significant reduction in PML-NBs (Figure 7A, B) and a subsequent reduction in APBs in SaOS2 tumors (Figure 7A, C), indicating that As_2_O_3_ treatment disrupts APB formation in mouse xenografts *in vivo*. In addition, we examined whether ALT activity is abolished upon As_2_O_3_ administration. We utilized the native FISH assay to detect single-stranded C-rich telomeric DNA (ssTeloC), such as C-circles, which are features of ALT activity (19,54,55). Our findings demonstrate that As_2_O_3_ treatment led to a reduction of ssTeloC in SaOS2 xenografts (Figure 7D, E), suggesting a loss of ALT activity. Together, these data suggest that As_2_O_3_ may represent a potential ALT inhibitor via targeting APB formation. This offers preliminary support for its future preclinical/clinical development aimed at exploring its efficacy in ALT cancer treatment.

## Discussion

Telomeric association of orphan NRs has been suggested to regulate the ALT pathway (36–40). In the present study, by tethering orphan NRs to telomeres, we observed ALT induction in human fibroblasts. Then we explored the role of orphan NRs in ALT induction using this *in vitro* system. Our results reveal that the telomeric localization of orphan NRs induces the formation of APBs and telomere clustering, which subsequently promotes ALT telomere recombination as evidenced by C-circle formation and ALT telomere DNA synthesis. PML knockout from human fibroblasts abolished ALT telomere DNA synthesis, supporting the critical role of APBs in orphan NR-induced ALT activation. These roles of orphan NRs are also consistent in ALT cell lines. Depletion of orphan NR COUP-TF2 from U2OS and WI38-VA13/2RA cells diminished APB formation, as well as ALT telomere DNA synthesis, supporting a dependency on orphan NRs for the ALT activation in ALT cells. Similarly, the ALT telomere DNA synthesis in ALT cells was inhibited upon silencing their PML expression. These findings are in agreement with previous studies (7,19) and further highlight a critical role for PML/APBs in mediating ALT activation in cancers via the telomeric association of orphan NRs.

Our system in which orphan NRs are tethered to telomeres for ALT induction in human fibroblasts has enabled us to dissect the mechanism directly contributing to orphan NR-mediated ALT activation. It has been shown previously that orphan NRs bind to the variant TCAGGG repeats of telomeres in ALT cells and they recruit the NuRD-ZNF827 complex, which may alter chromatin structures by impeding shelterin binding and histone deacetylase-mediated hypoacetylation to promote telomere recombination (37,38). Similarly, we found that ZNF827 is critical for ALT induction by orphan NRs in human fibroblasts, consistent with the notion that chromatin alteration may underlie orphan NR-induced ALT activation. Interestingly, we showed that orphan NR-mediated APB formation in human fibroblasts is also regulated by ZNF827, but it remains to be determined if this is also a consequence of changes in chromatin structure. It has also been shown previously that the orphan NRs COUP-TF2 and TR4 directly interact with FANCD2 to induce a DNA damage response and to contribute to the ALT pathway in ALT cells (39). However, our results show that depletion of FANCD2 does not affect APB formation, telomere clustering, and telomere DNA synthesis in fibroblasts. It is worth noting that FANCD2 has also been shown to restrain ALT activity by resolving replication stress at ALT telomeres, with data supporting that depletion of FANCD2 leads to more ALT activity (56). This discrepancy in the role of FANCD2 from three studies highlights the complexity of the function of FANCD2 in ALT activation, suggesting the necessity for additional investigation. Particularly, factors such as DDR activation at telomeres and the levels of FANCD2 expression may influence the role of FANCD2 in ALT induction. Our differing results from previous studies may arise due to the following factors: the use of different cell models—specifically, human fibroblasts versus U2OS and WI38-VA13/2RA ALT cells; the absence of detectable telomeric DDR in primary fibroblasts upon orphan NR-induced ALT (Supplementary Figure S3), contrasting with findings in ALT cells; varying levels of FANCD2 expression between non-transformed fibroblasts and ALT cell lines; and differences in methodology for FANCD2 depletion (RNAi vs. CRISPR). Moreover, the MMS21-SMC5-SMC6 SUMO E3 ligase complex has been shown to promote APB formation in ALT cells via sumoylation of TRF1 and TRF2 (15). Similar to FANCD2, MMS21-SMC5-SMC6 complex is not required APB formation and telomere clustering our system. Thus, these proteins do not seem to play direct roles in orphan NR-mediated ALT activation. The discrepancy observed from different studies may again be due to cell line-dependent roles of these proteins or experimental methodologies. Nevertheless, we do not rule out the possibility that these proteins may promote maintenance and stabilization of APBs and telomere clusters in ALT cells.

It has been shown previously that ALT cells exhibit spontaneous telomere DDRs, likely arising from replication stress or telomere trimming (11). Thus, DDR-related proteins may be preferentially recruited to and activated on ALT telomeres to modulate ALT activities. In this study, we present ALT induction by orphan NRs in non-transformed BJ^T^ cells, as well as in three additional primary cell lines BJ, IMR90, and WI38, which have intact p16 and p53. Remarkably, these primary cells expressing COUP-TF2^LBD^-TRF1 exhibited sustained normal cell growth in culture, suggesting that intact p16 and p53 do not prevent ALT induction in primary fibroblasts (Supplementary Figure S2). Our results demonstrate that DNA damage was not detected upon tethering orphan NRs to human primary fibroblasts (Supplementary Figure S3), implying that ALT activation by orphan NRs in primary cells might occur even in the absence of telomere DNA damage responses. Transient DDR has been reported to occur at functional telomeres in the G2 phase of the cell cycle after DNA replication, enabling the formation of the t-loop structure (57). Accordingly, we anticipate that clustered telomeres with altered chromatin structures may engage with the intrinsic telomere DDR in APBs to initiate ALT recombination for subsequent ALT induction. This scenario may explain how the telomeric localization of orphan NRs promotes ALT induction in human fibroblasts in the absence of profound DDR.

Recently, several studies employing divergent strategies have reported induction of ALT phenotypes and activity in cultured cells (6,18,35,37,47,58,59). By incorporating the variant telomeric sequence TCAGGG or TGAGGG into telomeres, various ALT characteristics—including telomere dysfunction-induced foci (TIFs), C-circles, and heterogenous telomeres—have been induced in HT1080 sarcoma cells, albeit doing so was insufficient to trigger ALT telomere recombination (37). Directly inducing APB formation (18,58) or telomere double strand breaks (6) promotes ALT activity in ALT cell lines, but not in cells that utilize telomerase for telomere maintenance. Depletion of the ASF1 histone chaperone to perturb DNA replication elicited ALT phenotypes and telomere recombination in HeLa and immortalized (by hTERT and HPV E6-E7) human fibroblasts (35). These systems have been used for the elucidation of the ALT mechanism, but they have some caveats. For instance, these models often induce severe genomic and/or telomeric DNA damage, so they have to be established in cancer cell lines (telomerase or ALT) or transformed primary cells that are defective in cell cycle checkpoints. Selectively activating particular ALT features, e.g. APBs, in ALT cells promotes higher ALT activity, but they cannot trigger ALT induction in non-ALT cells (18,58). In contrast, our system for ALT induction in human fibroblasts circumvents these limitations. First, tethering orphan NRs to telomeres did not trigger a significant telomere DDR in human fibroblasts, so the cells could proliferate normally. Targeting orphan NRs to telomeres triggered ALT phenotypes and ALT telomere DNA synthesis in ALT cells, as well as in primary human fibroblasts, indicating that orphan NRs coordinate multiple pathways for ALT induction. Consistently, the features of ALT activity we observed was dependent on both ZNF827 and PML, so ALT induction may result from the cooperative effects of the structural changes in chromatin and APB formation following telomeric targeting of orphan NRs. Thus, our system represents a unique *in vitro* strategy for ALT activation and can be deployed to investigate the ALT mechanism and ALT cancer development.

Our findings suggest that orphan NRs can promote telomere clustering upon their localization to telomeres in human fibroblasts. In the absence of PML, orphan NR-mediated telomere clustering was partly reduced in human fibroblasts, an outcome consistent with a previous finding showing that APB formation promotes telomere clustering (18,47). Intriguingly, we still detected substantial telomere clustering in PML knockout human fibroblasts, implying that the telomere clustering induced by orphan NRs can occur in an APB-independent manner. However, unlike for APB formation and ALT telomere DNA synthesis, telomere clustering in ALT cells was not affected by overexpression or knockdown of orphan NRs (Figure 2, Supplementary Figure S4). Moreover, telomere clustering in ALT cells was not affected upon depletion of PML, although it did abolish ALT telomere DNA synthesis. These results indicate that telomere clustering in ALT cells may occur independently of orphan NR functions and APB formation. It would be intriguing to further elucidate the underlying mechanism of telomere clustering in ALT cells, which is critical for understanding its role in ALT activation. Moreover, future work is also required to investigate the cell functions of orphan NR-mediated telomere clustering.

**Figure 8.**
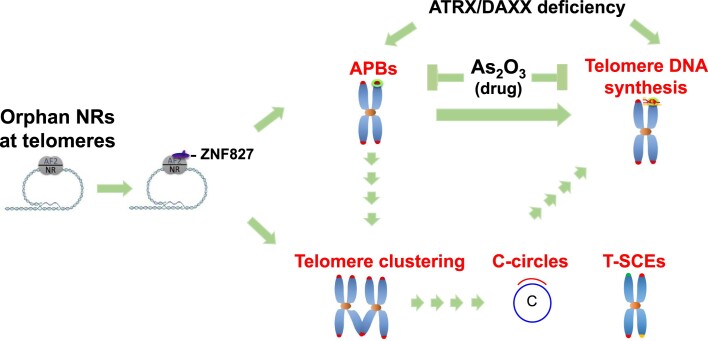
Model of orphan NR-induced ALT activation. Orphan NRs bind to ALT telomeres via variant repeats. The orphan NRs at telomeres recruit ZNF827 and allow for APB formation, telomere clustering, C-circle formation, telomere sister chromatid exchange, and telomere DNA synthesis. The telomere localization of orphan NRs acts in concert with ATRX/DAXX loss to promote APB formation and telomere DNA synthesis. APBs are critical for orphan NR-mediated ALT induction. Arsenic trioxide is an ALT inhibitor that can target APBs and features of ALT activity. Solid arrows indicate that the relationship between ALT phenotypes is established by this study. Broken arrows indicate an undetermined causal relationship between ALT phenotypes.

ATRX/DAXX mutations are the most frequently reported genetic alterations associated with ALT. However, it has been shown previously that ATRX depletion from mortal or telomerase-positive immortal cells is insufficient to activate ALT (31). Instead, ATRX loss progressively changes telomere chromatin structures and induces telomere dysfunction and cell growth arrest (34). We have demonstrated herein that combinatorial orphan NR targeting to telomeres and depletion of ATRX/DAXX induced ALT DNA synthesis in human fibroblasts (Figure 4), supporting that orphan NR recruitment to telomeres and ATRX/DAXX dysfunction represent two triggering events that may cooperatively promote ALT activation (Figure 8). Consistently, ATRX/DAXX depletion and telomeric targeting of orphan NRs elicited additive effects of APB formation and telomere clustering. However, ATRX/DAXX loss can also lead to telomere decompaction (34). Additionally, we observed that orphan NRs stimulate ALT phenotypes through ZNF827 (Figure 3), which has been shown to enhance chromatin compaction through the NuRD complex (38). However, it remains to be determined how chromatin compaction is altered under our experimental settings. Nevertheless, our findings suggest that the effects of telomere compaction upon ATRX/DAXX dysfunction and telomere recruitment of orphan NRs do not counteract ALT activation. In addition to telomere compaction, ATRX/DAXX loss has been implicated in promoting replication stress to stimulate ALT by increasing the expression of telomeric long noncoding RNAs (TERRA, telomeric repeat-containing RNA), R-loops (33,60,61) and G-quadruplex structures (32,62), as well as promoting loss of telomere cohesion (63,64), and dynamic exchange of histone macroH2A1.2 (65). Further comprehensive study is required to understand how ATRX dysfunction-associated changes interplay with orphan NRs to regulate ALT. It is worth noting that although ATRX/DAXX is a chaperone complex for telomeric deposition of histone H3.3, we did not observe changes in ALT phenotypes upon knockdown of histone H3.3 by siRNAs in control human fibroblasts or those expressing TRF1-orphan NRs, suggesting that transient depletion of histone H3.3 does not alter telomere chromatin structures. This outcome supports that dysregulation of the non-chaperone function of ATRX/DAXX can mediate ALT activation. However, we cannot rule out the possibility that prolonged ATRX/DAXX dysfunction can result in decreased histone H3.3 deposition at telomeres to promote ALT activation. Furthermore, while we observed induction of multiple features of ALT activity upon tethering of orphan NRs to telomeres in fibroblasts, this was not sufficient to counteract the effects of replication-associated telomere shortening and directly result in cell immortalization (unpublished data). Establishing whether the telomeric localization of orphan NRs promotes telomere lengthening upon ATRX/DAXX loss warrants further investigation.

Ultimately, we have demonstrated that APBs are critical for orphan NR-mediated ALT induction of human fibroblast cells and for enhancing features of ALT activity in ALT cells. APBs are sites of homologous recombination and are critical for ALT activity (7,8,19,66). We and others (7,19) have shown that depletion of PML from ALT cell lines abolishes ALT telomeric DNA synthesis. Significantly, PML is required for the ALT induction in our primary fibroblast cells based ALT model, and depletion of orphan NRs from ALT cells limits APB formation and ALT telomere DNA synthesis. It has been postulated previously that PML is important in ALT because it localizes the BLM-TOP3A-RMI (BTR) complex to ALT telomeres (19). Identifying that PML is critical for orphan NR-mediated ALT induction led us to explore if targeting APBs or PML is a potential anti-ALT strategy. Arsenic trioxide, which promotes PML degradation, has long been used as an APL treatment and, recently, it has been repurposed for other cancer types such as osteosarcoma and glioblastoma (51,52). Here, we demonstrate that arsenic trioxide can act as a potential ALT inhibitor given that it degrades PML to prevent APB formation and ALT telomere DNA synthesis in both primary fibroblasts and ALT cell lines. Aside from our *in vitro* experiments, we extended our investigation to demonstrate that arsenic trioxide also disrupts APBs and ssTeloC in mouse xenografts. Although multiple compounds have been investigated for ALT targeting, these studies have predominantly been conducted in cultured cells, serving primarily as proof of concept (44,67). Nevertheless, our study demonstrates for the first time that a clinically approved drug can inhibit features of ALT activity *in vivo*. Consequently, we propose a novel anti-ALT cancer strategy involving repurposing arsenic trioxide, which could undergo further clinical development for ALT cancer patients. Overall, our significant findings illuminate the role of orphan NRs in ALT cancer development and provide valuable information that may be utilized for ALT cancer treatment.

## Supplementary Material

gkae389_Supplemental_File

## Data Availability

The data underlying this article are available in the article and in its online supplementary material. Further data underlying this article will be shared on reasonable request to the corresponding author.
