# Supplementary material for: Orphan nuclear receptors-induced ALT-associated PML bodies are targets for ALT inhibition
Source: Nucleic Acids Res. 2024 May 16;52(11):6472–89. doi: 10.1093/nar/gkae389 (PMC11194075; doi:10.1093/nar/gkae389)
Supplement: gkae389_Supplemental_File [file gkae389_supplemental_file.pdf]

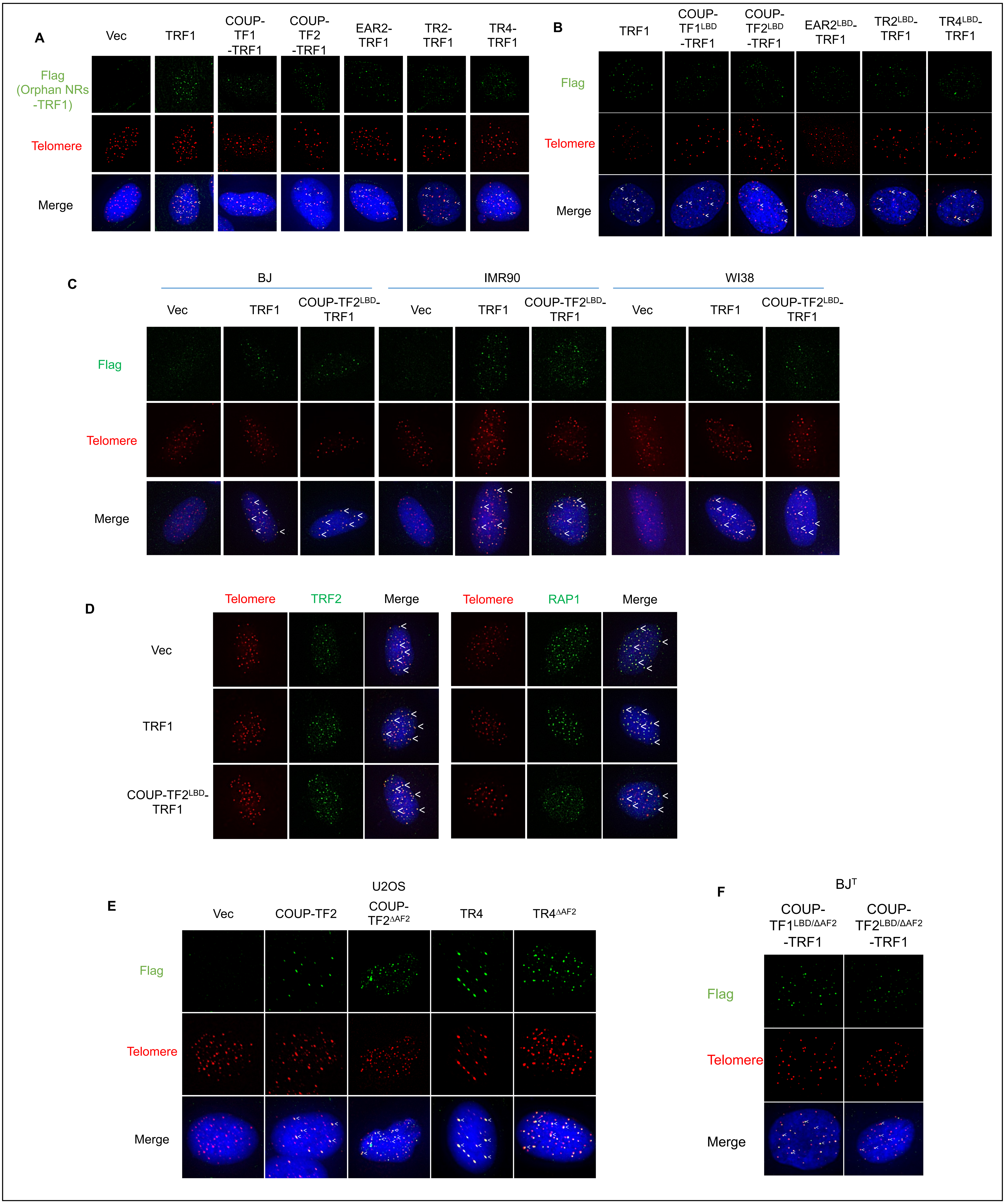

Figure S1. Subcellular localization of orphan NR tethering to telomeres. (A) Representative images showing co-localization of Flag-tagged COUP-TF1-TRF1, COUP-TF2-TRF1, EAR-TRF1, TR2-TRF1, and TR4-TRF1 fusion proteins with telomeres in BJ^{T} cells. **(B)** Representative images showing co-localization of Flag-tagged COUP-$TF1^{LBD}$-TRF1, COUP-$TF2^{LBD}$-TRF1, $EAR2^{LBD}$-TRF1, $TR2^{LBD}$-TRF1, and $TR4^{LBD}$-TRF1 fusion proteins with telomeres in BJ^{T} cells. **(C)** Representative images showing co-localization of Flag-tagged COUP-$TF2^{LBD}$-TRF1 fusion proteins in primary BJ, IMR90, and WI38 cells. **(D)** Representative images showing co-localization of shelterin components TRF2 and RAP1 to telomeres in BJ^{T}-COUP-$TF2^{LBD}$-TRF1 cells. **(E)** Representative images showing co-localization of Flag-tagged COUP-TF2, COUP-$TF2^{\Delta AF2}$, TR4, and $TR4^{AF2}$ with telomeres in U2OS cells. **(F)** Representative images showing co-localization of Flag-tagged COUP-$TF1^{LBD/\Delta AF2}$-TRF1 and COUP-$TF2^{LBD/\Delta AF2}$-TRF1 fusion proteins with telomeres. The Flag tag was detected by immunofluorescence (IF), and telomeres were detected by FISH using the TelC PNA probe. Co-localization of Flag (green) and telomeres (red) appears yellow. White arrows indicate co-localization of Flag-tagged proteins and telomeres.

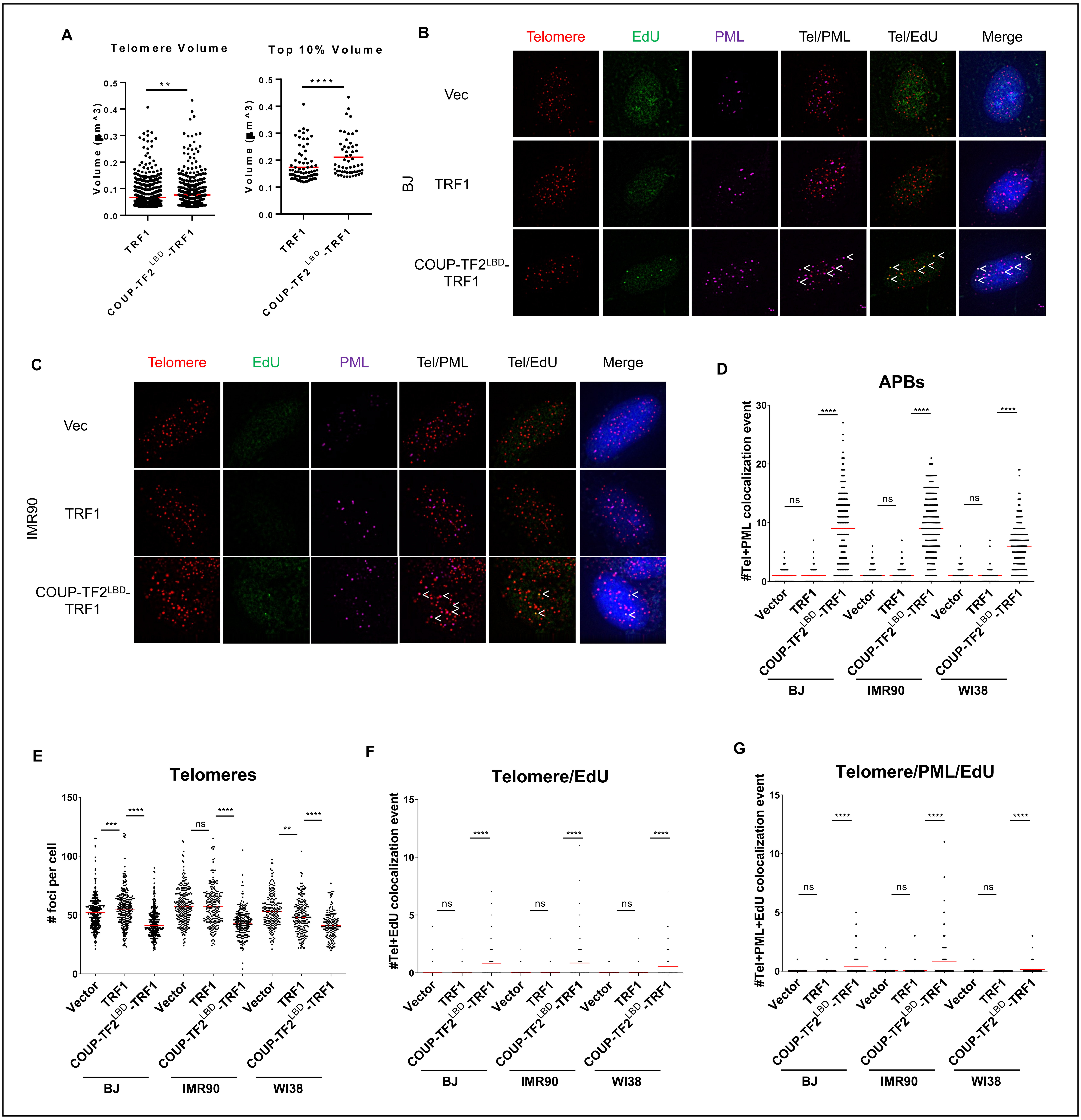

Figure S2. Tethering of orphan NRs to telomeres induces ALT in fibroblasts. (A) Quantification of telomere volume in BJ^{T}-COUP-TF2^{LBD}-TRF1 cells. Representative images showing PML and telomere co-localization and EdU and telomere co-localization in BJ **(B)** and IMR90 **(C)** cells expressing the LBD of the orphan NR COUP-TF2 fused with TRF1. EdU and PML were detected by IF, and telomeres were detected by FISH using the TelC PNA probe. Co-localization of EdU (green), PML (magenta), and telomeres (red) appears white. White arrows indicate telomeric DNA synthesis at APBs. Quantification of APBs **(D)**, telomere numbers **(E)**, telomere and EdU co-localization **(F)**, and telomere, PML, and EdU co-localization **(G)** in individual BJ, IMR90, and WI38 cells (n>100) expressing LBD of the orphan NR COUP-TF2 fused with TRF1. Red lines represent median/mean of two independent experiments. ns p>0.05, *p<0.05 **p<0.01, ***p<0.001, ****p<0.0001, as determined by Mann-Whitney U test.

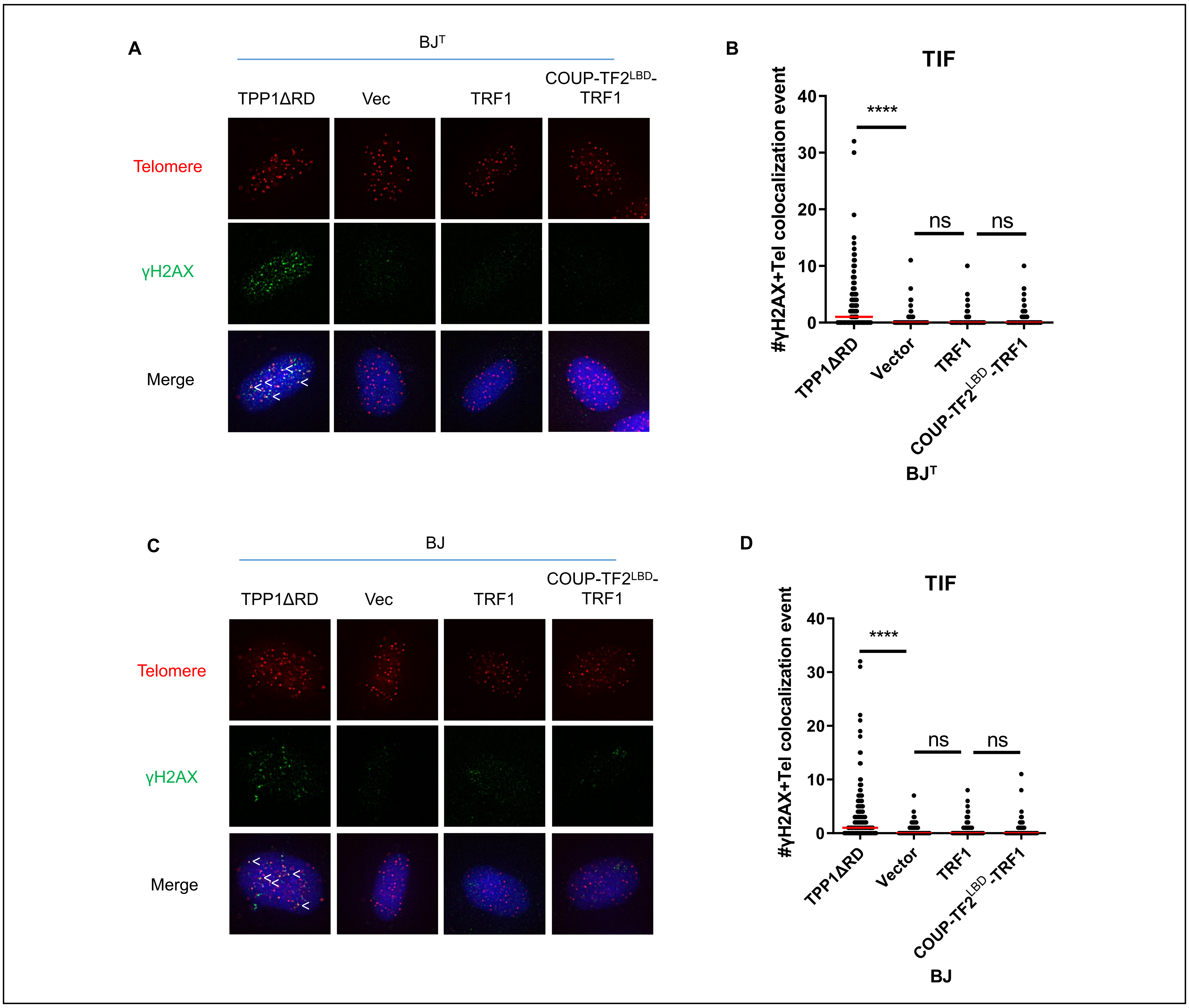

Figure S3. DNA damage is not detected upon orphan NR-mediated ALT induction in fibroblasts. (A) Representative images showing non-co-localization of γH2AX and telomeres in BJ^{T}-COUP-TF2^{LBD}-TRF1 cells. γH2AX was detected by IF and telomeres were detected by FISH using the TelC PNA probe. Co-localization of γH2AX (green) and telomeres (red) yellow. White arrows indicate telomere dysfunction-induced foci (TIFs) in positive control BJ^{T}-TPP1ΔRD cells. **(B)** Quantification of TIFs in BJ^{T} cells (n>100). **(C)** Representative images showing non-co-localization of γH2AX and telomeres in primary BJ-COUP-TF2^{LBD}-TRF1 cells. γH2AX was detected by IF and telomeres were detected by FISH using the TelC PNA probe. Co-localization of γH2AX (green) and telomeres (red) yellow. White arrows indicate telomere dysfunction-induced foci (TIFs) in positive control primary BJ-TPP1ΔRD cells. **(D)** Quantification of TIFs in primary BJ cells (n>100). Red lines represent median. ns $p>0.05$, *$p<0.05$ **$p<0.01$, ***$p<0.001$, ****$p<0.0001$, as determined by Mann-Whitney U test.

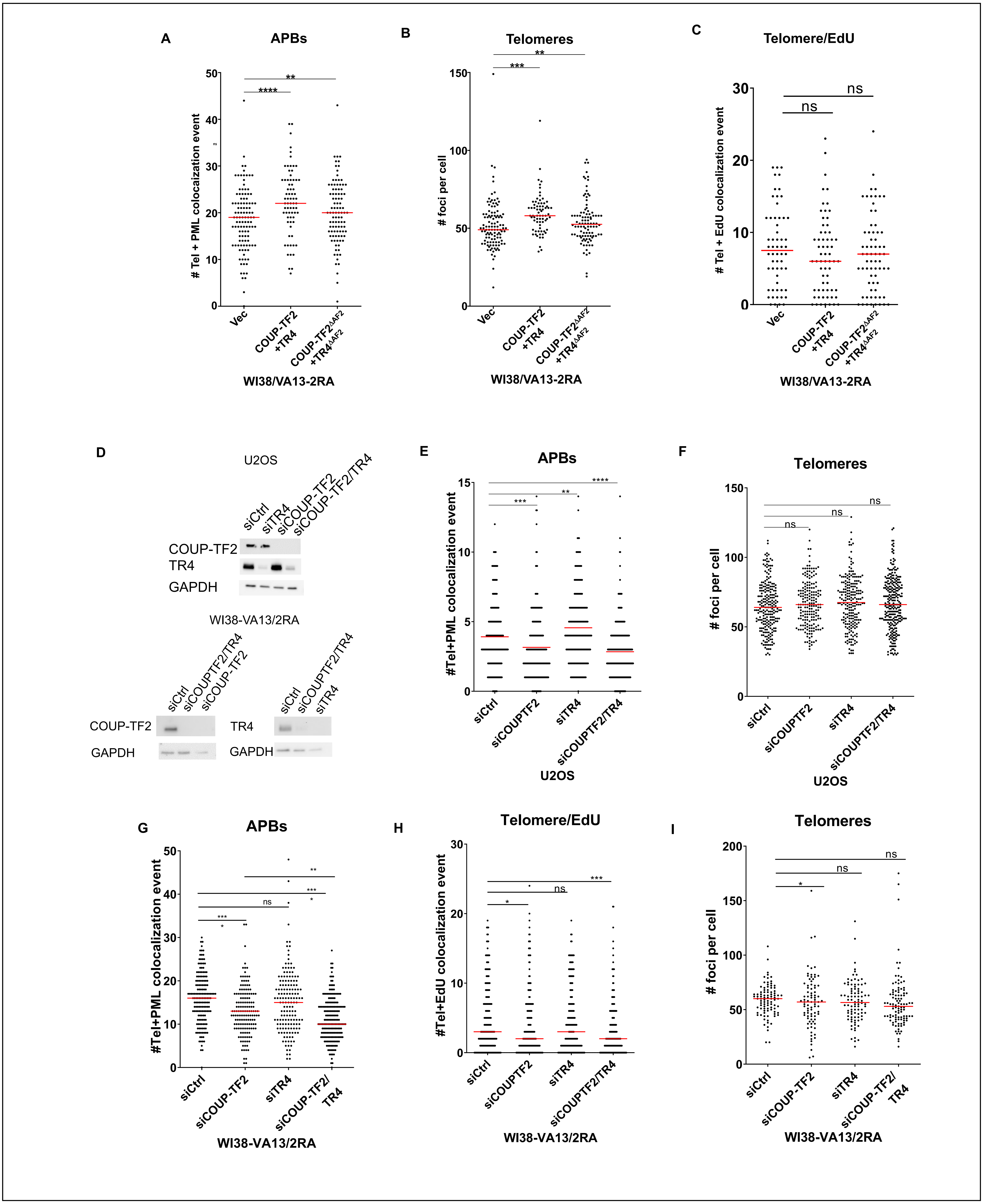

Figure S4. Orphan NRs mediate ALT activation in ALT cells. Quantification of APBs **(A)**, telomere numbers **(B)**, and telomere and EdU co-localization **(C)** in individual WI38-VA13/2RA cells (n>100) expressing the orphan NRs COUP-TF2 and TR4 or COUP-TF2$^{\Delta AF2}$ and TR4$^{\Delta AF2}$. **(D)** Western blot showing COUP-TF2 and TR4 expression in U2OS and WI38-VA13/2RA cells. Quantification of APBs **(E)** and telomere numbers **(F)** in U2OS cells upon treatment with siRNAs against COUP-TF2 or TR4. Quantification of APBs **(G)**, telomere and EdU co-localization **(H)**, and telomere numbers **(I)** in WI38-VA13/2RA cells upon treatment with siRNAs against COUP-TF2 or TR4. Red lines represent median/mean. ns $p>0.05$, *$p<0.05$ **$p<0.01$, ***$p<0.001$, ****$p<0.0001$, as determined by Mann-Whitney U test.

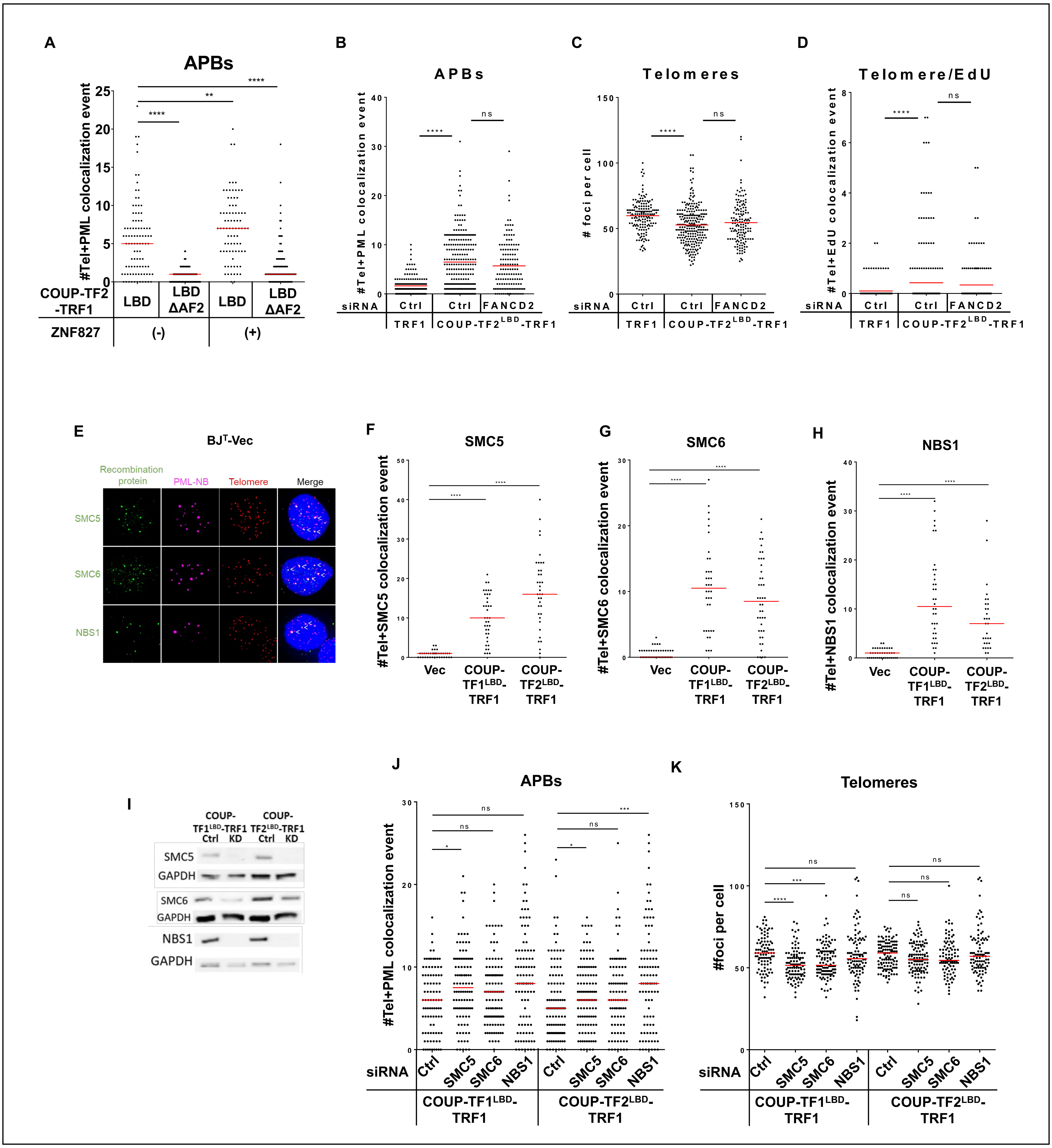

Figure S5. Recombination proteins are not critical for orphan NR-mediated ALT induction. (A) Quantification of APBs in individual BJ^{T}-COUP-$TF2^{LBD}$-TRF1 cells and BJ^{T}-COUP-$TF2^{LBD/\Delta AF2}$-TRF1 cells with or without ZNF827 overexpression (n>100). Quantification of APBs **(B)**, telomere numbers **(C)**, and telomere and EdU co-localization **(D)** in individual BJ^{T}-COUP-$TF2^{LBD}$-TRF1 cells (n>100) upon treatment with siRNAs against FANCD2. **(E)** Representative images showing co-localization of PML-NBs and recombination proteins before APB formation in BJ^{T}-Vec cells. Recombination proteins (SMC5, SMC6, and NBS1) and PML/DAXX were detected by IF, and telomeres were detected by FISH using the TelC PNA probe. Co-localization of recombination proteins (green) and PML/DAXX (magenta) appears white. White arrows indicate co-localization of recombination proteins and PML-NBs in BJ^{T} cells. Quantification of the co-localization of recombination proteins SMC5 **(F)**, SMC6 **(G)**, and NBS1 **(H)** with telomeres in BJ^{T}-COUP-$TF1^{LBD}$-TRF1 and BJ^{T}-COUP-$TF2^{LBD}$-TRF1 cells (n>100). **(I)** Western blot showing reduced protein expression in BJ^{T}-COUP-$TF1^{LBD}$-TRF1 and BJ^{T}-COUP-$TF2^{LBD}$-TRF1 cells upon treatment with siRNAs against SMC5, SMC6, or NBS1. Quantification of APBs **(J)** and telomere numbers **(K)** in individual BJ^{T}-COUP-$TF1^{LBD}$-TRF1 and BJ^{T}-COUP-$TF2^{LBD}$-TRF1 cells (n>100) upon treatment with siRNAs against SMC5, SMC6, or NBS1. Red lines indicate median/mean. ns $p>0.05$, *$p<0.05$ **$p<0.01$, ***$p<0.001$, ****$p<0.0001$, as determined by Mann-Whitney U test or unpaired t-test.

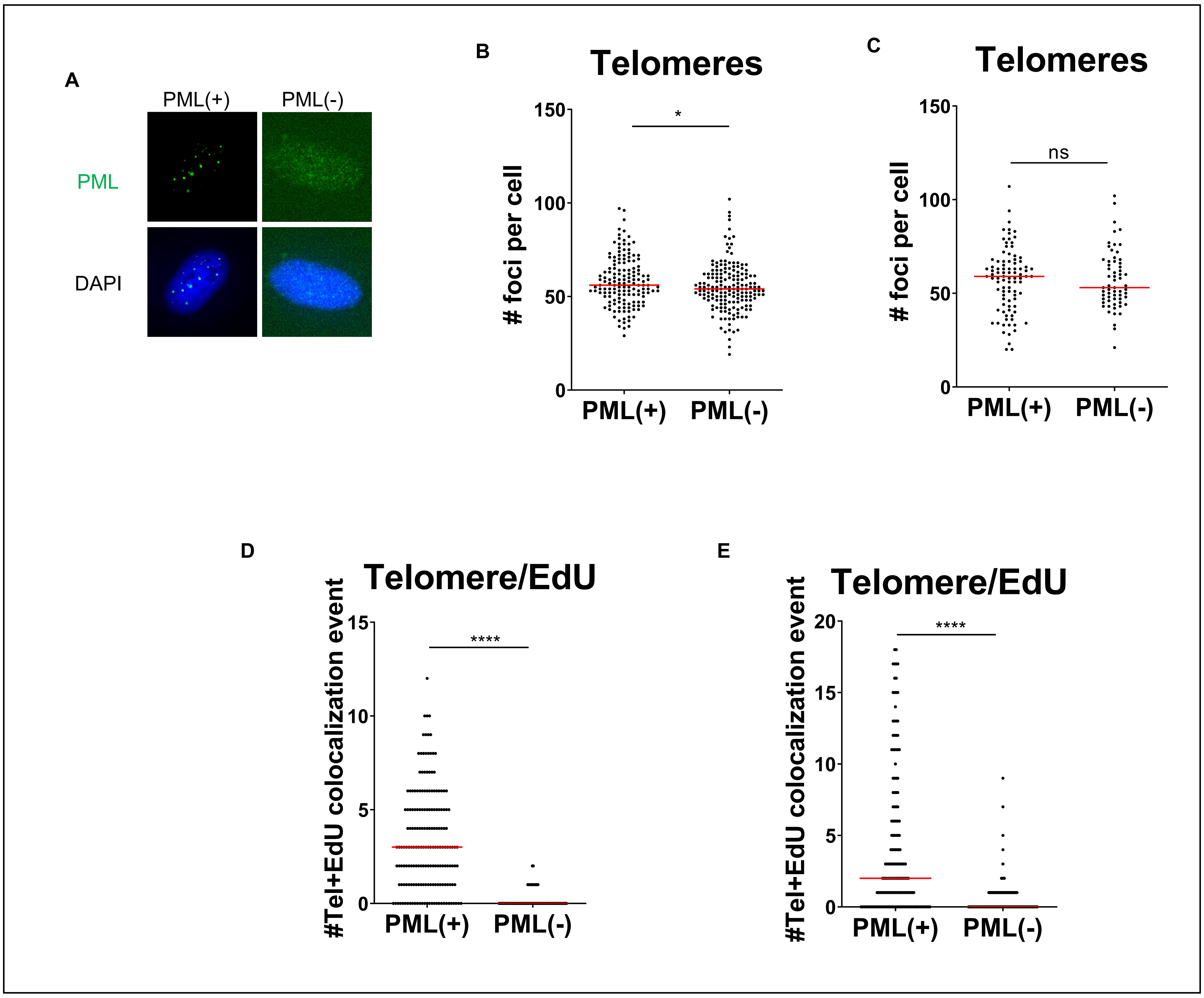

Figure S6. PML is critical for telomeric DNA synthesis at APBs. (A) Representative images of PML(+) and PML(-) U2OS cells. PML was detected by IF. Quantification of telomere numbers in PML(+) and PML(-) U2OS **(B)** and WI38-VA13/2RA **(C)** cells (n>100). Quantification of telomere and EdU co-localization in PML(+) and PML(-) U2OS **(D)** and WI38-VA13/2RA **(E)** cells (n>100). Cells were synchronized in G2 phase by means of thymidine and CDK1i treatments for 21 h and 12 h, respectively. Red lines represent median of two independent experiments. ns $p>0.05$, *$p<0.05$ **$p<0.01$, ***$p<0.001$, ****$p<0.0001$, as determined by Mann-Whitney U test.

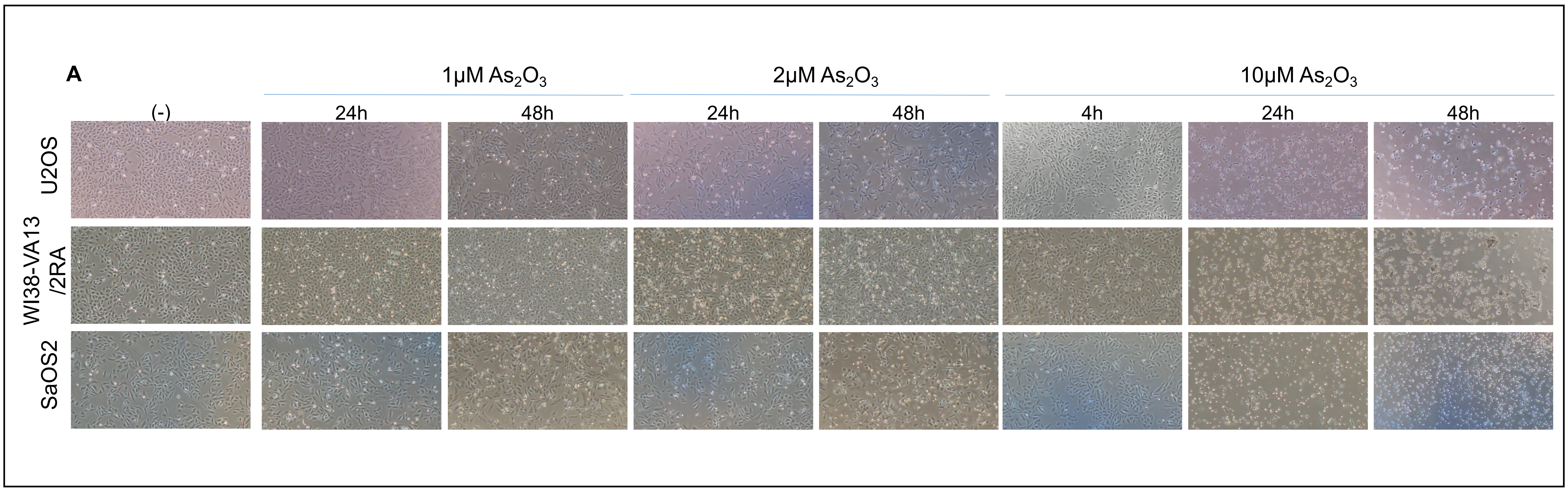

Figure S7. Arsenic trioxide cytotoxicity in ALT cells.

(A) Cytotoxicity assay showing 1 μM As_2O_3 as the sublethal concentration in U2OS, WI38-VA13/2RA, and SaOS2 cells.
